# Combination therapy protects macaques against advanced Marburg virus disease

**DOI:** 10.1038/s41467-021-22132-0

**Published:** 2021-03-25

**Authors:** Robert W. Cross, Zachary A. Bornholdt, Abhishek N. Prasad, Viktoriya Borisevich, Krystle N. Agans, Daniel J. Deer, Dafna M. Abelson, Do H. Kim, William S. Shestowsky, Lioudmila A. Campbell, Elaine Bunyan, Joan B. Geisbert, Karla A. Fenton, Larry Zeitlin, Danielle P. Porter, Thomas W. Geisbert

**Affiliations:** 1 Galveston National Laboratory, University of Texas Medical Branch, 301 University Blvd, Galveston, TX USA; 2 Department of Microbiology and Immunology, University of Texas Medical Branch, 301 University Blvd, Galveston, TX USA; 3 Mapp Biopharmaceutical, Inc., 6160 Lusk Blvd Ste C200, San Diego, CA USA; 4 Gilead Sciences, Inc., 333 Lakeside Dr, Foster City, CA USA

**Keywords:** Viral infection, Preclinical research

## Abstract

Monoclonal antibodies (mAbs) and remdesivir, a small-molecule antiviral, are promising monotherapies for many viruses, including members of the genera *Marburgvirus* and *Ebolavirus* (family *Filoviridae*), and more recently, SARS-CoV-2. One of the major challenges of acute viral infections is the treatment of advanced disease. Thus, extending the window of therapeutic intervention is critical. Here, we explore the benefit of combination therapy with a mAb and remdesivir in a non-human primate model of Marburg virus (MARV) disease. While rhesus monkeys are protected against lethal infection when treatment with either a human mAb (MR186-YTE; 100%), or remdesivir (80%), is initiated 5 days post-inoculation (dpi) with MARV, no animals survive when either treatment is initiated alone beginning 6 dpi. However, by combining MR186-YTE with remdesivir beginning 6 dpi, significant protection (80%) is achieved, thereby extending the therapeutic window. These results suggest value in exploring combination therapy in patients presenting with advanced filovirus disease.

## Abstract (web summary)

Extending the therapeutic window for acute viral infections could save lives. Here, the authors show that combination treatment with a human monoclonal antibody and remdesivir initiated at 6 days post infection with Marburg virus provides 80% protection in non-human primates.

## Introduction

Members of the genera *Marburgvirus* and *Ebolavirus* are human pathogens in the family *Filoviridae* that are responsible for outbreaks of severe and highly fatal hemorrhagic disease. Marburg virus (MARV) outbreaks are initiated through contact by humans with the only known reservoir host of the virus, the Egyptian rousette bat (*Rousettus aegyptiacus*)^1,2^ or its excreta, and are further amplified and sustained by human-to-human transmission. Outbreaks of MARV in human populations are usually limited in size and duration, sometimes involving only a single clinical case, but have also occurred explosively over extended durations with large numbers of cases and very high case fatality rates (CFR). The latter scenario is exemplified by the 1998–2000 outbreak in the Democratic Republic of Congo (128 deaths/153 cases, 84% CFR)^3^, and the 2004–2005 outbreak in Angola (227 deaths/252 cases, 90% CFR)^4^. The timing of outbreaks in humans has been correlated with seasonal spikes in the prevalence of the virus in bat populations^5^. Surveillance studies in *R. aegyptiacus* have implied a substantial, albeit fragmented geographic distribution of the virus throughout sub-Saharan Africa^6–8^. However, MARV has been identified in rousette populations from areas with no known incidences of human infection^7,9–11^. Thus, outbreaks of MARV in human populations remain difficult, if not impossible, to predict. In addition, weaponized MARV is considered a high priority bioterrorism threat^12,13^. Therefore, in addition to preventative measures such as vaccines, the development and validation of effective postexposure therapies for MARV disease (MVD) is of critical importance to facilitate rapid deployment upon identification of MARV cases.

Much of the research into MARV-specific postexposure treatments has centered upon the development of neutralizing and non-neutralizing antibody-based therapies targeting the MARV glycoprotein (GP)^1,14–20^. Additionally, small-interfering RNAs (siRNAs) targeting MARV messenger RNAs (mRNA) have been designed and evaluated in nonhuman primate (NHP) models, with survival conferred when treatment is initiated up to 5 days post-infection (dpi)^21,22^. Of several developed approaches for the discovery of monoclonal antibodies (mAbs)^23^, the most successful approach with regard to infectious agents appears to be the identification and isolation of memory B-cells producing agent-specific mAbs from the blood of survivors^24–27^. With regard to MARV, this approach has yielded many high-value mAb candidates, such as the MARV neutralizing mAbs MR186 and MR191, which directly target the receptor binding site on MARV GP and have been extensively evaluated in vitro and in vivo^16–18,20,28^. Of significance, MR191-N, produced using a *Nicotiana benthamiana* transient plant expression system^29^, rescued rhesus macaques from lethal infection when administered as two 50 mg/kg doses beginning as late as 5 dpi^17^.

In addition to MARV-specific mAbs, broad spectrum small-molecule antivirals have been investigated for potential activity against MARV infection in vivo. Remdesivir (GS-5734) is a monophosphoramidate prodrug of an adenosine nucleoside analog, with demonstrated inhibitory activity in NHPs against several diverse lineages of RNA viruses, including members of *Filoviridae*^30,31^, *Paramyxoviridae*^32^, and *Coronaviridae*^33,34^. Remdesivir is currently the only antiviral therapy for the treatment of COVID-19^35^ with demonstrated clinical benefit in multiple Phase 3 trials of hospitalized patients^36–38^ to receive marketing approval from by the US Food and Drug Administration^39^. Remdesivir inhibits viral replication by impeding synthesis of viral RNA by the viral RNA-dependent RNA polymerase via delayed chain termination as well as template-mediated inhibition mechanisms^40,41^.

Here, we investigated the therapeutic efficacy of a half-life extended version of the MARV mAb MR186 (MR186-YTE, the afucosylated form which promotes antibody-dependent cellular cytotoxicity and neutrophil activation/phagocytosis^42^) in a single 100 mg/kg dose and a once daily remdesivir dosing regimen in a uniformly lethal rhesus macaque model of MARV variant Angola infection. We then evaluated the therapeutic benefit of co-administering these treatments during advanced stages of MARV disease at a point beyond successful therapeutic intervention for either product alone. Our findings indicate that therapeutics with complementary mechanisms of action can be combined to extend the window of therapeutic intervention and may serve to inform future efforts to combat MARV outbreaks as well as the general treatment of emerging viruses in human populations.

## Results

### Experimental challenge of rhesus macaques with MARV and therapeutic rescue from lethal MVD

We first assessed the therapeutic efficacies of MR186-YTE and remdesivir in treating MARV infection individually. MR186 was selected as a therapeutic candidate based on previously reported in vitro data^16^ and modified with Fc mutations to increase the serum half-life of the antibody.

**Fig. 1:**
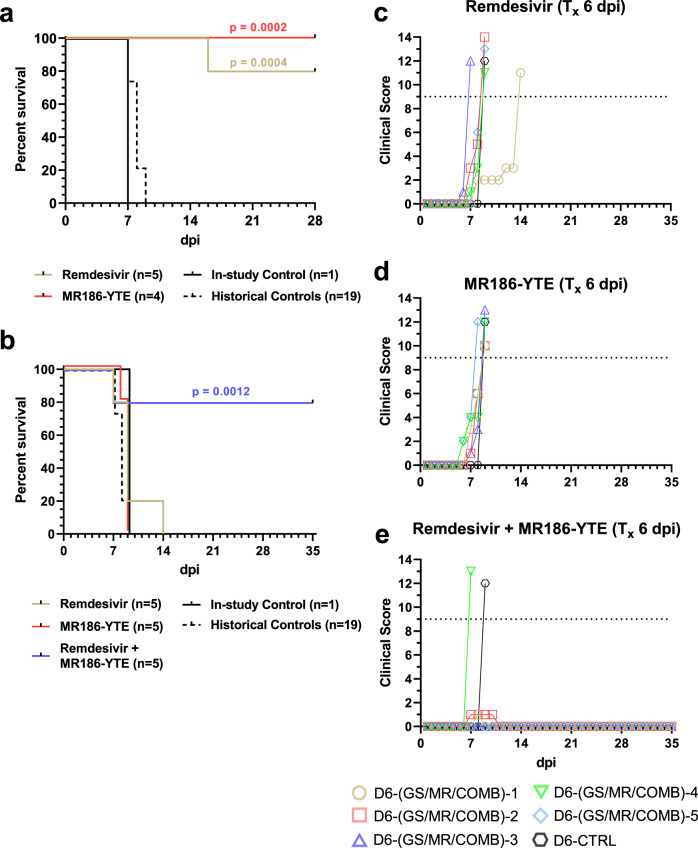
Survival analysis and clinical scoring of rhesus macaques challenged with MARV virus. **a** Kaplan–Meier survival curves of rhesus macaques receiving treatment beginning 5 dpi and untreated and historical control animals. **b** Kaplan–Meier survival curves of rhesus macaques receiving treatment beginning 6 dpi and untreated and historical control animals. For (**a**, **b**), curves for the in-study control and historical control animals are shown separately; however, for statistical comparison, the in-study control was pooled with the historical controls. The reported two-tailed *p* value was derived from Fisher’s exact test corrected for multiple comparisons using the Hochberg method, and was rounded to four decimal places. **c**–**e** Clinical scoring for rhesus macaques with treatment initiated 6 dpi with remdesivir (**c**), MR186-YTE (**d**), and combined remdesivir/MR186-YTE (**e**). The horizontal dashed line represents the minimum clinical score by which euthanasia criteria was met. T_x_ = “treatment”. Source data are provided as a Source Data file.

We conducted an experiment where ten healthy, adult rhesus macaques were challenged intramuscularly (i.m.) with a target dose of 1000 PFU of MARV (Angola variant). At 5 dpi, one cohort of animals (*n* = 5) received a 10 mg/kg intravenous (i.v.) loading dose of remdesivir, followed by 5 mg/kg daily maintenance doses 6–16 dpi, for a total of 12 consecutive days of treatment, as previously described^31^. A second cohort (*n* = 4) received a single 100 mg/kg i.v. dose of MR186-YTE 5 dpi. A single animal was treated with vehicle-only and served as the in-study positive control. The vehicle-treated control animal developed symptoms of MVD beginning 5 dpi and succumbed 7 dpi, consistent with the mean time to death (MTD) for historical controls (HC) challenged i.m. with the same seed stock and target dose of MARV (MTD = 7.95 ± 0.71 dpi; *n* = 19). All animals in the remdesivir-treated group developed mild to moderate clinical disease consistent with MVD, beginning with the appearance of fever 5–8 dpi and including decreased appetite/anorexia, petechial rash, and diarrhea in some or all animals (Supplementary Table 1 and Supplementary Fig. 1a), and 4/5 animals survived to the study endpoint (28 dpi) (Fig. 1a). All surviving animals were free of overt signs of MVD by 19 dpi. These results are consistent with a previous study demonstrating the efficacy of remdesivir in a cynomolgous macaque model of MARV disease^31^. Subjects in the MR186-YTE treated cohort exhibited considerably lower clinical scores (Supplementary Fig. 1b) and milder clinical signs of MVD than the control animal, primarily consisting of fever (Supplementary Table 1) and decreased appetite; a single animal (D5-MR-3) exhibited sparse petechial skin rash on 8 and 9 dpi (Supplementary Table 1). All subjects in this group were free of clinical signs by 16 dpi and all subjects survived to the study endpoint (28 dpi) demonstrating the effectiveness of MR186-YTE as a single dose therapeutic for the treatment of MVD (Fig. 1a). For statistical comparisons of survival, the in-study control animal was grouped with the 19 HC animals (*n* = 20, MTD = 7.90 ± 0.72 dpi). There was a significant difference in survival between the remdesivir treated group and the control cohort (Hochberg multiplicity-corrected *p* = 0.0004; Fisher’s exact test), and between the MR186-YTE treated group and the control cohort (Hochberg multiplicity-corrected *p* = 0.0002; Fisher’s exact test). All subjects regardless of treatment exhibited deviation from baseline hematological and serum analyte parameters, including lymphocytopenia, generalized granulocytosis (indicated by increased neutrophils with or without concurrent increases in eosinophils and basophils), thrombocytopenia, and increases in markers of liver injury (e.g., ALT, AST, GGT, ALP) and systemic inflammation (CRP) (Supplementary Table 1 and Supplementary Fig. 2). These changes were not markedly different between treated animals and the control animal prior to the initiation of treatment except for CRP, which was increased in the control animal compared to most animals from both treated groups at 5 dpi. Animals from both treatment groups exhibited a gradual return to baseline values for most parameters 8–14 dpi, indicating recovery. In summary, each therapeutic was demonstrated to be highly efficacious for treatment of MARV infected rhesus macaques on 5 dpi under their respective dosing regimen.

### Combining therapeutics with complementary mechanisms of action enhances protective efficacy and extends the window of treatment for lethal MVD

Given the results of the previous experiment and the clear therapeutic benefit observed when either treatment was initiated 5 dpi, we next conducted a study with 16 adult rhesus macaques utilizing the same seed stock and challenge dose of MARV/Angola variant as the previous study, but where initiation of treatment was delayed to 6 dpi. In addition to remdesivir and MR186-YTE-only treatment groups, the study design included a third cohort of animals treated with a single dose of MR186-YTE 6 dpi in tandem with a 12-day course of remdesivir treatment (6–17 dpi). A single untreated animal served as the in-study positive control. Similar to the previous study, the control animal developed clinical signs of MVD beginning 6 dpi, including fever, decreased appetite/anorexia, jaundiced appearance, and petechial rash; and succumbed to disease 9 dpi (Supplementary Table 2). Similarly, subjects in the remdesivir and MR186-YTE-only treated groups displayed signs of MVD beginning 6 dpi. However, in stark contrast to the animals treated on 5 dpi, all animals from both the remdesivir and MR186-YTE treated groups succumbed to disease before the study endpoint (Fig. 1b) (MTD = 9.60 ± 2.61 and 8.80 ± 0.45 dpi, respectively), and in the case of the remdesivir-treated group, prior to the completion of the treatment schedule. In contrast, 4/5 animals (80%) in the combined remdesivir/MR186-YTE treatment group survived to the study endpoint (35 dpi). As in the previous study, for statistical comparisons of survival, the in-study control from this study was grouped with 19 HC animals (*n* = 20, MTD = 8.00 ± 0.73 dpi). Statistically significant differences in survival between the treatment groups and controls were only detected for animals receiving combined remdesivir/MR186-YTE treatment (Hochberg multiplicity-corrected *p* = 0.0012; Fisher’s exact test). Subjects in the combined treatment cohort exhibited lower clinical scores (Fig. 1c–e) and a rapid reduction in fever that was present at the initiation of treatment (Supplementary Table 2). One of the surviving animals in the combination treatment group (D6-COMB-2) exhibited mild petechiation covering less than 20% of the body 7–10 dpi (Supplementary Table 2). The subject that succumbed from this group (D6-COMB-4) developed severe clinical signs of MVD beginning on the day that treatment was initiated and succumbed to disease the following day (7 dpi). As in the first study, all animals exhibited marked deviation from baseline hematological and serum analyte values at 6 dpi (Supplementary Table 2 and Supplementary Fig. 3). The severity of disease in the remdesivir and MR186-YTE monotherapy-treated groups increased until subjects reached euthanasia criteria. Remarkably, despite treatment being delayed by one day compared to the 5 dpi monotherapy study, the four surviving animals which received combination therapy recovered to baseline levels of leukocytes, thrombocytes, and renal/hepatic function markers more rapidly than animals receiving either treatment alone at 5 dpi, with most parameters reaching normal or near-normal values by 12 dpi.

### Therapeutic reduction of viral load

All animals from the single agent 5 dpi treatment initiation study and combination 6 dpi treatment initiation study were viremic on the day treatment was initiated, and there was no significant difference in the amount of circulating infectious virus between treatment groups and the respective in-study control pooled with HC sampled at the same time point, as determined by one-way ANOVA followed by Tukey’s post-hoc test (Supplementary Fig. 4; *p* ≥ 0.1081 for all comparisons). Viremia began to decline following initiation of treatment but continued to rise in the control animal up to the terminal timepoint (Supplementary Fig. 5a–d). Animals treated with MR186-YTE were free of detectable viral genomic RNA (vRNA) in whole blood sooner than surviving animals treated with remdesivir (mean dpi of last positive RT-qPCR result = 10.25 ± 1.5 and 13.25 ± 1.5, respectively). Viral load was assessed by qPCR from tissues harvested at necropsy from all animals (Supplementary Fig. 5e–g). MARV vRNA was detected in all tissues tested from some or all animals in the remdesivir-treated group; but was absent or below the limit of detection (LOD) in certain tissues (e.g., adrenal gland, gonad, conjunctiva) from all animals in the MR186-YTE-treated group. vRNA copy number equivalents were ~2–4 log_10_ lower in matched tissues from subject D5-GS-4 (succumbed 16 dpi), versus the control animal (succumbed 7 dpi) (Supplementary Fig. 5g). Infectious virus was not recovered from eye or splenic tissue harvested from selected animals receiving either treatment and surviving to the study endpoint.

**Fig. 2:**
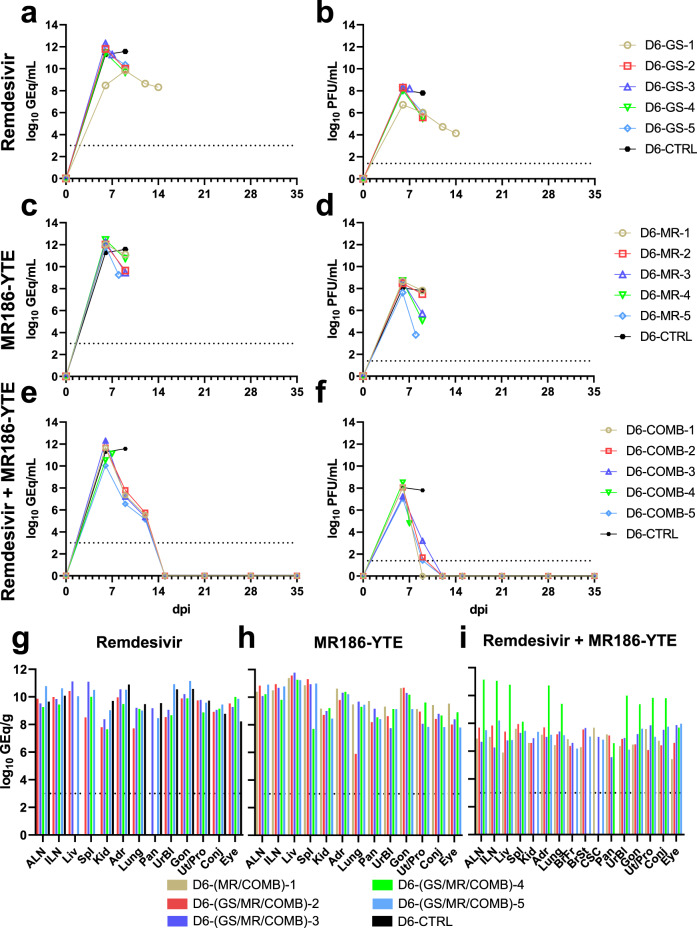
Viral load in blood and tissues of MARV-challenged rhesus macaques receiving therapeutic treatment beginning 6 dpi. Viral load was determined by RT-qPCR of whole blood (**a, c, e**) or selected tissues harvested at necropsy (**g**–**i**), or plaque titration of plasma (**b**, **d**, **f**). For all panels, individual data points represent the mean of two technical replicates. Dashed horizontal lines indicate the limit of detection (LOD) for the assay (1000 GEq/mL for RT-qPCR; 25 PFU/mL for plaque titration). To fit on a log scale axis, zero values (below LOD) are plotted as “1” (10^0^). Abbreviations for tissues: ALN axillary lymph node, ILN inguinal lymph node, Liv liver, Spl spleen, Kid kidney, Adr adrenal gland, Pan pancreas, UrBl urinary bladder, Gon gonad, Ut/Pro uterus/prostate, Conj conjunctiva. Source data are provided as a Source Data file.

When either remdesivir or MR186-YTE treatment was administered alone starting 6 dpi, reductions in viral load were less apparent, and animals continued to be viremic to the point they met euthanasia criteria (Fig. 2a–d). Conversely, animals receiving combination treatment rapidly cleared the virus, and all surviving animals in this cohort were free of detectable circulating vRNA and infectious virus by 15 dpi (mean dpi of last positive RT-qPCR result = 12 ± 0 dpi) (Fig. 2e, f). All animals regardless of treatment group had detectable MARV vRNA in most or all tissues (Fig. 2g–i). Subject D6-COMB-4, which succumbed to infection 7 dpi, had comparable levels of vRNA in tissues to animals in both individual treatment groups, which was markedly higher than what was observed in tissues from surviving combination treated animals (Fig. 2i). We were unable to isolate infectious virus from any tissues collected from the surviving combination treated animals at the study endpoint, indicating that the detected vRNA in these animals was a residual artifact of a cleared infection. Subject D6-GS-1, which succumbed to infection 14 dpi, was positive in all tissues from an expanded collection panel, including the CNS and other immunologically privileged sites (Supplementary Fig. 6a). Infectious virus was recovered from these tissues, confirming the presence of replicating virus at the time of death (Supplementary Fig. 6b); however, this animal did not exhibit any noticeable signs of neurological involvement (Supplementary Table 2).

### Gross and histopathology

Necropsy was performed on all animals following euthanasia. Among monkeys that succumbed to disease, lesions were consistent with previous descriptions of MARV in this model^43^, regardless of treatment (13/14 animals, including in-study controls). The most frequent lesions were petechial rash, diffuse hepatic pallor with rounded edges, splenomegaly, lymphadenomegaly, and abdominal effusion. Subject D5-GS-4, which was treated with remdesivir and succumbed to disease at 16 dpi in the 5 dpi monotherapy study, lacked classic gross lesions of MARV infection, with only mild disseminated lymphadenomegaly. All animals that survived to the study end point lacked gross lesions.

**Fig. 3:**
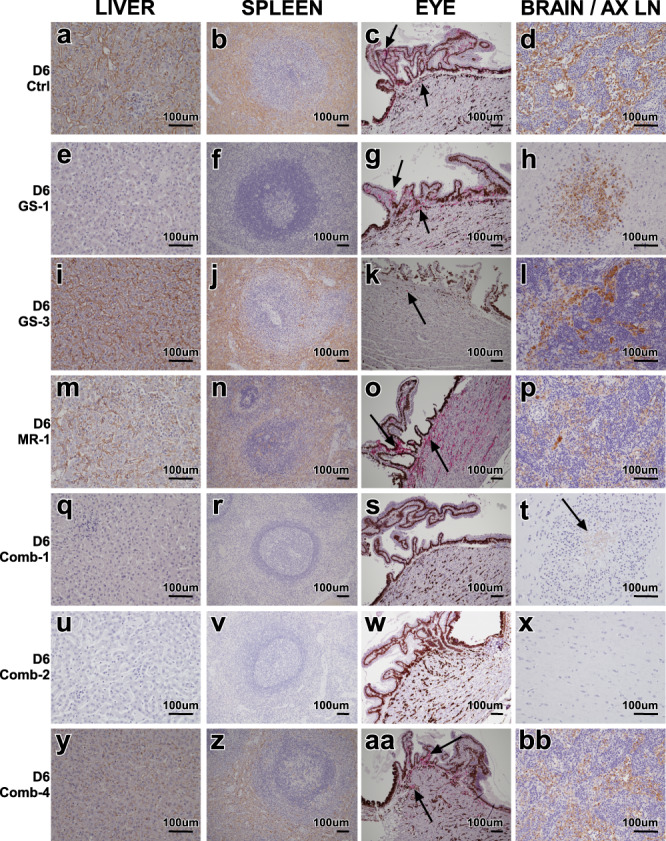
Histologic changes in rhesus macaques infected with MARV with and without treatment. **a–d** Tissues of positive control rhesus D6-Ctrl. MARV IHC positive (brown) **a** sinusoidal lining cells of the liver and Kupffer cells, **b** mononuclear cells in the splenic red and white pulp, **c** MARV IHC positive (red) epithelial and mononuclear cells within the ciliary body of the eye (arrows), **d** MARV IHC positive (brown) of mononuclear cells within the sinuses of the axillary lymph node. **e**–**h** Tissues of remdesivir treated rhesus D6-GS-1 that succumbed to disease 14 dpi. **e** No appreciable MARV IHC immunolabeling of the liver, **f** No appreciable MARV IHC immunolabeling of the spleen, **g** MARV IHC positive (red) epithelial and mononuclear cells within the ciliary body of the eye (arrows), **d** MARV IHC positive (brown) of glial cells within brain. **i–l** Tissues of remdesivir treated rhesus D6-GS-3 that succumbed to disease 7 dpi. MARV IHC positive (brown) **i** sinusoidal lining cells of the liver and Kupffer cells, **j** mononuclear cells in the splenic red and white pulp, **k** MARV IHC positive (red) mononuclear cells within the ciliary body of the eye (arrow), **l** MARV IHC positive (brown) of mononuclear cells within the sinuses of the axillary lymph node. **m**–**p** Tissues of MR186-YTE treated rhesus D6-MR-1 that succumbed to disease 9 dpi. MARV IHC positive (brown) **m** sinusoidal lining cells of the liver and Kupffer cells, **n** mononuclear cells in the splenic red and white pulp, **o** MARV IHC positive (red) epithelial and mononuclear cells within the ciliary body of the eye (arrows), **p** MARV IHC positive (brown) of mononuclear cells within the sinuses of the axillary lymph node. **q**–**t** Tissues of combination remdesivir and MR186-YTE treated rhesus D6-Comb-1 that succumbed to disease 14 dpi. **q** No appreciable MARV IHC immunolabeling of the liver, **r** No appreciable MARV IHC immunolabeling of the spleen, **s** No appreciable MARV IHC immunolabeling (red) of the eye, **t** MARV IHC positive (brown) of glial cells within brain (arrow). **u**–**x** Tissues of combination remdesivir and MR186-YTE treated rhesus D6-Comb-2 that survived to study end point. **u** No appreciable MARV IHC immunolabeling of the liver, **v** No appreciable MARV IHC immunolabeling of the spleen, **w** No appreciable MARV IHC immunolabeling (red) of the eye, **x** No appreciable MARV IHC immunolabeling of the brain. **y**–**bb** Tissues of combination remdesivir and/ MR186-YTE treated rhesus D6-Comb-4 that succumbed to disease 7 dpi. MARV IHC positive (brown) **y** sinusoidal lining cells of the liver and Kupffer cells, **z** mononuclear cells in the splenic red and white pulp, **aa** MARV IHC positive (red) epithelial and mononuclear cells within the ciliary body of the eye (arrows), **bb** MARV IHC positive (brown) of mononuclear cells within the sinuses of the axillary lymph node. Images captured at 20x (**a**, **e**, **i**, **m**, **q**, **u**, **y**, **d**, **h**, **l**, **p**, **t**, **x**, & **bb**) 10x (**b**, **f**, **j**, **n**, **r**, **v**, **z**, **c**, **g**, **k**, **o**, **s**, **w**, & **aa**) IHC labeling for anti-MARV antigen (brown) (**a**, **b**, **d**, **e**, **f**, **h**, **i**, **j**, **l**, **m**, **n**, **p**, **q**, **r**, **t**, **u**, **v**, **x**, **y**, **z**, & **bb**) IHC labeling for anti-MARV antigen (red) (**c, g, k, o, s, w,** & **aa**). For each animal, displayed images are representative of histopathological lesions and immunoreactivity observed in multiple tissue slices and/or fields of view, unless otherwise indicated.

Histologic findings corroborated the gross findings in monkeys that succumbed to MARV infection (Fig. 3). Microscopic lesions associated with MARV-infection noted in these monkeys included, but were not limited to: necrotizing hepatitis with sinusoidal leukocytosis, hepatocellular cytoplasmic inclusion bodies, splenic lymphoid depletions with fibrin expansion of the red pulp (Fig. 3b), sinusoidal histocytosis of lymph nodes (Fig. 3d), mild uveitis, and mild interstitial pneumonia. Mixed inflammation was also variably noted in kidney, adrenal gland, pancreas, urinary bladder, gonads, uterus or prostate, and conjunctiva. Immunohistochemical (IHC) labeling for MARV antigen in monkeys that succumbed to disease co-localized with lesions noted on H&E staining, consistent with previous observations^43^. Subjects D6-GS-1 and D5-GS-4, which succumbed to disease at 14 and 16 dpi, respectively, had similar microscopic lesions; however, they were less pronounced than those that succumbed during the acute phase, and both subjects had encephalitis with co-localized IHC positive glial cells (Fig. 3h). Microscopic evaluation of animals surviving to the study end point did not reveal histologic lesions typical of MARV infection; however, mild multifocal lymphocytic infiltrates in the liver (12/12 animals) (Fig. 3a, e, i, m, q, u, y) and kidney (2/12 animals) were noted. Few IHC positive mononuclear cells within the anterior uvea were noted in all monkeys treated with MR186-YTE in the first study. Two monkeys from the remdesivir/MR186-YTE combination treatment cohort had lesions within the brain, encephalitis with co-localized IHC positive glial cells (subject D6-COMB-1, Fig. 3t), and lymphocytic perivascular cuffs (subjects D6-COMB-1 and D6-COMB-3). All other tissues collected at the study end point, regardless of treatment, were unremarkable for H&E and IHC. Severity scores as assigned by a board-certified veterinary pathologist are provided (Table S3 and S4).

## Discussion

The continuing outbreaks of emerging and re-emerging viral diseases, currently exemplified by the ongoing COVID-19 pandemic, emphasize the need for effective vaccines and therapeutics. Although the two largest MARV outbreaks exhibited extremely high (~80–90%) CFRs, they have occurred with less regularity and have resulted in far fewer overall cases and fatalities than Ebola virus (EBOV)^44^. However, the prevalence of the virus in a species of bat with very large, geographically disperse populations in Africa, and anthropogenic factors which favor viral emergence (e.g. habitat destruction, hunting and consumption of wild animals), cast an ominous portent for future outbreaks. Additionally, the potential for deliberate misuse by rogue actors or nation states should not be discounted^12,13^.

MAbs and broad spectrum small molecule antivirals have been at the forefront as candidates for addressing these viral outbreaks and have been evaluated during the last two filovirus outbreaks and are currently being studied during the SARS-CoV-2 pandemic. A clinical trial conducted during the tail end of the 2013–2016 West African EBOV epidemic, and terminated early due to the end of the outbreak, found that a mAb cocktail, ZMapp, was 92% likely to have been superior to standard of care in reducing mortality^45^. Subsequently, during the 2018–2019 DRC EBOV outbreak, the PALM trial found that two other mAb products, mAb114 and REGN-E3, were statistically superior to ZMapp and remdesivir at reducing mortality from EBOV disease^46^. Additionally, in October 2020, REGN-COV2, remdesivir, and dexamethasone were used in combination under compassionate use criteria in a human a who had tested positive for and was experiencing symptoms of SARS-CoV-2 infection^47^. Subsequently, after demonstrating positive results in treating hospitalized COVID-19 patients in Phase 3 clinical trials, remdesivir was granted marketing approval by the US FDA^35^.

A recent study of MARV (Angola variant) infection and therapeutic rescue in cynomolgus macaques found a statistically significant survival benefit (83%) when a 10 mg/kg loading dose of remdesivir administered beginning on 5 dpi was followed by daily 5 mg/kg maintenance doses for a total of 12 days^31^. Here, we expanded upon those findings by assessing the therapeutic efficacy of remdesivir and the MARV-specific mAb MR186-YTE in rhesus macaques, alone and in combination, up to 6 dpi. The results of our studies define a therapeutic window for MVD for these monotherapies, where treatment with remdesivir or MR186-YTE alone initiated 5 dpi resulted in rescue from lethal MVD in 4/5 and 4/4 animals, respectively, but uniformly failed to protect animals from lethal infection when administered 6 dpi. Conversely, combined treatment with both remdesivir and MR186-YTE was able to rescue most animals from late stage disease (MTD = 8.0 dpi), with 4/5 animals surviving infection with a rapid reversal of observable signs of clinical illness and reduction of circulating virus in the peripheral blood. The one animal that was not rescued, subject D6-COMB-4, succumbed 7 dpi. This animal did not exhibit a higher viral load or more severe perturbations to hematological parameters than other subjects in the cohort at 6 dpi, when treatment was initiated; however, marked lymphadenopathy, liver pallor, splenomegaly, adrenomegaly, and edema with hemorrhage along the dorsal wall of the body cavity were observed at necropsy, indicating this animal may have been closer to the terminal phase of disease when treatment was initiated. While MARV vRNA was still detected in the tissues of animals surviving to the study endpoint, we were unable to isolate infectious virus from these animals, including from potential immunologically privileged reservoir sites, indicating complete clearance of the virus following convalescence. A limitation of this second study is that we did not determine the upper limit to where delayed combination treatment with MRE186-YTE and remdesivir confers significant rescue in macaques from fatal MVD. However, the studies presented here provide a framework for future efforts to define the therapeutic window of efficacy for combined mAb and remdesivir treatment.

Assessing the efficacy of candidate postexposure therapeutics in treating acute infectious disease in humans inherently presents numerous logistical challenges. With regard to filoviruses, these challenges are compounded by the severity of the disease as well as sociopolitical, economic, and cultural factors in their endemic regions. Moreover, variability between patients in exposure dosage, portal of entry by the virus, and most importantly, the severity of illness present when treatment is administered, all likely play significant roles in determining responsiveness to treatment and overall clinical outcome.

Due to the non-descript nature (“flu-like symptoms”) of the early presentation of filovirus disease in humans, many patients do not seek medical care until the more severe clinical manifestations of the disease (e.g., emesis, abdominal pain, maculopapular rash, coagulopathy with internal and/or external hemorrhage) become apparent. At this stage, patients may already be experiencing severe and possibly irreversible multi-organ damage and subsequent failure. In the PALM trial, patients were enrolled an average of 5.5 days after first presenting with symptoms of EBOV disease, and 97% of the deaths in the trial occurred within 10 days of enrollment^46^. Treatment with remdesivir or ZMapp was found to result in lower odds of survival in comparison to MAb114 (single mAb) or REGN-EB3 (cocktail of three mAbs); however, despite the clear survival benefit of both MAb114 and REGN-EB3 that resulted in a recent marketing approval of REGN-EB3 by the US FDA, the mortality in patients receiving these two mAb products was still 33–34%, underscoring further need for improvement of these treatment outcomes, potentially via deployment of a combination therapy. In addition, the study authors note the fact that the full treatment courses of MAb114 and REGN-EB3 were administered as a single dose, whereas remdesivir and ZMapp were administered over multiple doses, may play a role in the observed differences in survival benefit. This conclusion was supported by a faster rate of viral clearance from the blood in patients treated with MAb114 and REGN-EB3 compared to those treated with remdesivir or ZMapp (determined by RT-qPCR). Similarly, we observed a significantly faster viral clearance rate in surviving animals treated 5 dpi with MR186-YTE versus remdesivir when comparing the day of the last positive RT-qPCR result. Thus, our observation that coupling a single-dose mAb with a multi-day schedule of remdesivir extended the therapeutic window of efficacy by at least 1 day in the rhesus macaque model is likely to be translatable to improved treatment outcomes in humans suffering from advanced filovirus disease.

The additional benefit of the combination therapy likely stems from the complementary mechanisms of action between the small molecule viral replication inhibitor remdesivir and the neutralizing antibody MR-186-YTE. Remdesivir reduces both the expression of viral antigens and infectious virus release from infected cells and MR186-YTE protects cells and tissues from spreading infection and might also facilitate clearance of newly produced viral particles. In addition, because of their very different physico-chemical properties, the two therapeutics likely exhibit non-overlapping tissue distribution profiles and thus might block the virus spread more broadly across different body compartments and tissues.

We recently demonstrated that mAb therapy can augment vaccination prior to the development of protective immunity in a rhesus macaque model of EBOV infection^48^. Animals vaccinated with rVSV-ZEBOV 1 day prior to challenge with a lethal dose of EBOV and then treated 3 dpi with MIL77, a two-mAb cocktail, were completely rescued from the development of clinical illness and death, in contrast to animals receiving only vaccination or mAb therapy. Here, we have shown enhanced therapeutic benefit of combination mAb and remdesivir treatment of MARV infection. To our knowledge, this work is the first demonstration of significant rescue from lethal disease in an animal model of filovirus infection when treatment is initiated beyond 5 dpi. Taken together, these studies suggest that a “two-pronged” approach to filovirus postexposure prophylaxis/therapy may serve to mitigate the high case fatality rates associated with these viruses during outbreaks in humans. This study also suggests that mAbs in combination with small molecule antivirals with complementary mechanisms of action may potentially provide a treatment paradigm with increased therapeutic benefit for other acute viral diseases as well.

## Methods

### Virus

The MARV Angola seed stock originates from the 2004–2005 Uige, Angola outbreak (DQ: 447653.1). The virus was isolated from the serum of a patient who died from MVD on March 13, 2005 (8-month-old female; isolate 200501379). The study challenge material was created by passaging the original isolate in Vero E6 cells (passage 2 stock, titer 1.5 × 10^7^ PFU/mL). Endotoxin and mycoplasma contamination were not detected in stocks.

### NHP challenge and treatment

Details of the study design for each experiment are provided in the Results section of the manuscript. All procedures involving physical manipulation were performed under sedation with ketamine. For the first study, ten healthy, adult rhesus macaques (*Macaca mulatta*) of Chinese origin ranging in age from ~ 3–5 years and weighing 4.7–5.3 kg were challenged i.m. in the left or right quadricep with a 1000 PFU target dose (actual dose 1075 PFU) of MARV (Angola variant). Assignment to each treatment group or control was determined prior to challenge by randomization with effort made to maintain a balanced sex ratio. Treatment was initiated 5 dpi as either a single dose (MR186-YTE) or multi-dose (remdesivir) protocol. The duration of this study was 28 days. The second study involved 16 healthy, adult rhesus macaques ranging in age from ~ 3–5 years and weighing 3.5–5.6 kg, and was performed identically to the first study with exception to actual dose of the virus inoculum (1481 PFU), the day treatment was first administered (6 dpi), and the duration of the study (35 days). The macaques were monitored daily and scored for disease progression with an internal MARV humane endpoint scoring sheet approved by the UTMB Institutional Animal Care and Use Committee (IACUC). The scoring changes measured from baseline included posture and activity level, attitude and behavior, food intake, respiration, and disease manifestations, such as visible rash, hemorrhage, ecchymosis, or flushed skin. A score of ≥9 indicated that an animal met the criteria for euthanasia.

### Ethics statement

Animal studies were performed in BSL-4 biocontainment at the University of Texas Medical Branch (UTMB) and approved by the UTMB Institutional Biosafety Committee (IBC) and IACUC. Animal research was conducted in compliance with UTMB IACUC, Animal Welfare Act, and other federal statutes and regulations relating to animals. The UTMB animal research facility is fully accredited by the Association for Assessment and Accreditation of Laboratory Animal Care and adhere to principles specified in the eighth edition of the Guide for the Care and Use of Laboratory Animals, National Research Council.

### Drug formulation

CHOK1-AF cells stably expressing the MR186-YTE mAbs were generated as previously described^42^. Briefly, a dual-promoter plasmid containing expression cassettes for the heavy and light chains of the target mAb was transfected into the CHOK1-AF cell line with selection under MSX containing medium beginning 24 h post transfection. Upon recovery of the cells (~3 weeks), the now enriched pool of CHOK1-AF cells expressing MR186-YTE was expanded for expression of MR186-YTE in BalanCD Growth A medium containing 50 μM MSX. The culture was placed in a shaking CO2 incubator set at 5% CO2 and 140 RPM and over the next 14 days the culture was expanded to support large scale production of MR186-YTE in multiple 5 L shake flasks (Thomson). The cultures were then maintained in batch-mode for 10 days. The supernatant was then clarified via filtration and subsequently sterile filtered (0.2 µm) into a 20 L bioprocess bag (Thermo Fisher). MR186-YTE was purified from the clarified supernatant using a GE (now Cytiva) MabSelect SuRe LX Protein A affinity chromatography column on an AKTA pure 150 M3 system. MR186-YTE was loaded onto the MabSelect column, washed with HyClone 1X PBS, then eluted using 0.1 M Acetic Acid containing 0.2 M L-Arginine, pH 3.3. The eluate was immediately neutralized with 2 M Tris base to pH ~7. The neutralized eluate was then diluted fivefold using WFI quality water and purified via GE (now Cytiva) Capto Q chromatography column in a flow-through mode for endotoxin and host-cell DNA removal. The Capto Q flow-through containing >96% of the MR186-YTE was diafiltered against the formulation buffer (20 mM Citrate, 10 mM Glycine, 8% Sucrose, pH 5.52) and concentrated to 41.8 mg/mL. Polysorbate-80 was added to 0.01% after the target mAb concentration was reached.

Remdesivir was synthesized at Gilead Sciences, Inc. The chemical identity and sample purity were established using NMR, HRMS, and HPLC analyses^30^. Small-molecule X-ray crystallographic coordinates and structure factor files have been deposited in the Cambridge Structural Database (http://www.ccdc.cam.ac.uk/); accession numbers have been supplied previously^30^. Remdesivir drug substance batch number 5734-BC-1P was solubilized in 12% sulfobutylether-β-cyclodextrin in water at pH 3.5 and matching vehicle solution was provided to UTMB for these studies.

### Hematology and serum biochemistry

Total white blood cell counts, white blood cell differentials, red blood cell counts, platelet counts, hematocrit values, total hemoglobin concentrations, mean cell volumes, mean corpuscular volumes, and mean corpuscular hemoglobin concentrations were analyzed from blood collected in tubes containing EDTA using a Vetscan HM5 laser based hematologic analyzer (Zoetis). Serum samples were tested for concentrations of albumin, amylase, alanine aminotransferase (ALT), aspartate aminotransferase (AST), alkaline phosphatase (ALP), blood urea nitrogen (BUN), calcium, creatinine (CRE), C-reactive protein (CRP), gamma-glutamyltransferase (GGT), glucose, total protein, and uric acid by using a Piccolo point-of-care analyzer and Biochemistry Panel Plus analyzer disks (Abaxis).

### RNA isolation from MARV-infected macaques

On procedure days, 100 μl of blood from K2-EDTA collection tubes was collected prior to centrifugation and was added to 600 μl of AVL viral lysis buffer with 6 μL carrier RNA (Qiagen) for RNA extraction. For tissues, approximately 100 mg was stored in 1 ml RNAlater (Qiagen) for at least 4 days for stabilization. RNAlater was completely removed, and tissues were homogenized in 600 μl RLT buffer and 1% betamercaptoethanol (Qiagen) in a 2 mL cryovial using a tissue lyser (Qiagen) and 0.2 mm ceramic beads. The tissues sampled included axillary and inguinal lymph nodes, liver, spleen, kidney, adrenal gland, lung, pancreas, urinary bladder, ovary or testis, and eye. All blood samples were inactivated in AVL viral lysis buffer, and tissue samples were homogenized and inactivated in RLT buffer prior to removal from the BSL-4 laboratory. Subsequently, RNA was isolated from blood using the QIAamp viral RNA kit (Qiagen), and from tissues using the RNeasy minikit (Qiagen) according to the manufacturer’s instructions supplied with each kit.

### Quantification of viral load

Primers sequences used for detection of MARV RNA are provided in Supplementary Table 5. MARV RNA was detected using the CFX96 detection system (BioRad Laboratories) in one-step probe RT–qPCR kits (Qiagen) with the following cycle conditions: 50 °C for 10 min, 95 °C for 10 s, and 40 cycles of 95 °C for 10 s and 59 °C for 30 s. Threshold cycle (CT) values representing MARV *NP* genomes were analyzed with CFX Maestro Software, and data are shown as genome equivalents (GEq). To create the GEq standard, RNA from MARV stocks was extracted, and the number of MARV *NP* genomes was calculated using Avogadro’s number and the molecular weight of the MARV genome. LOD was 1 × 10^3^ GEq/ml.

Virus titration was performed by plaque assay using Vero E6 cells (ATCC CRL-1586) from all plasma and tissue samples as previously described^49^. Briefly, increasing tenfold dilutions of the samples were adsorbed to Vero E6 cell monolayers in duplicate wells (200 μl) and overlaid with 0.8% agarose in 1x Eagles minimum essentials medium (MEM) with 5% FBS and 1% P/S. After 6 days incubation at 37 °C/5% CO_2_, neutral red stain was added and plaques were counted after 48 h incubation. The LOD for this assay was 25 PFU/mL.

### Histopathology and immunohistochemistry

Necropsy was performed on all subjects. Tissue samples of all major organs were collected for histopathologic and IHC examination, immersion-fixed in 10% neutral buffered formalin, and processed for histopathology, as previously described^22^. Briefly, tissue sections were deparaffinized and rehydrated through xylene and graded ethanols. Slides went through heat antigen retrieval in a steamer at 95 °C for 20 min in Sigma Citrate Buffer, pH6.0, 10x (Sigma Aldrich, St. Louis, MO). To block endogenous peroxidase activity, slides were treated with a 3% hydrogen peroxide and rinsed in distilled water. The tissue sections were processed for IHC using the Thermo Autostainer 360 (ThermoFisher, Kalamazoo, MI). Sequential 15 min incubations with avidin D and biotin solutions (Vector, Burlingame, CA) were performed to block endogenous biotin reactivity. For IHC examination, virus-specific immunoreactivity was detected using a mouse anti-MARV NP primary antibody (provided by USAMRIID, catalog BB06-BB01^50^) at a 1:4000 dilution for 60 mins. The secondary antibody used was biotinylated goat anti-mouse IgG (Vector Laboratories) at 1:200 dilution for 30 min, followed by incubation with horseradish peroxidase conjugated with streptavidin (Vector Laboratories) for 30 min. Tissue sections were processed using the Dako Autostainer. Slides were developed with diaminobenzidine chromogen (Dako) for 5 min and counterstained with hematoxylin for 30 sec. Relative severity scores for histological lesions/immunoreactivity were assigned by a board-certified veterinary pathologist. Representative photomicrographs were qualitatively considered to display lesions that were nominally or ordinally measured by masking of the veterinary pathologist post-examination and ranking lesions to satiate the study objectives, as previously established^51^. Additionally, thorough examination of the target tissues were performed multiple times in a timely manner to maintain interpretation consistency.

### Statistical analysis

For statistical comparisons, survival and viremia data from ten published^17,21,22,49^ and nine unpublished historical control rhesus macaques challenged via the same route with the same virus stock and dose were added to the in-study control cohorts. All statistical analyses were performed in GraphPad Prism v8.4.3, except for the Hochberg correction for multiple comparisons, which was performed in R v.4.0.2^52^ using the “p.adjust” function of the included stats v.4.0.2 package. Unless otherwise indicated, all reported *p* values are two tailed and rounded to four decimal places.

### Reporting summary

Further information on research design is available in the Nature Research Reporting Summary linked to this article.

## Supplementary information

Supplementary Information

Reporting Summary

## Data Availability

The datasets used and/or analyzed during the current study are available from the corresponding author, T.W.G., on reasonable request. Source data are provided with this paper.

## Source data

Source Data
