# Supplementary material for: Combination therapy protects macaques against advanced Marburg virus disease
Source: Nat Commun. 2021 Mar 25;12:1891. doi: 10.1038/s41467-021-22132-0 (PMC7994808; doi:10.1038/s41467-021-22132-0)
Supplement: Supplementary file 1 — Supplementary Information [file 41467_2021_22132_MOESM1_ESM.pdf]

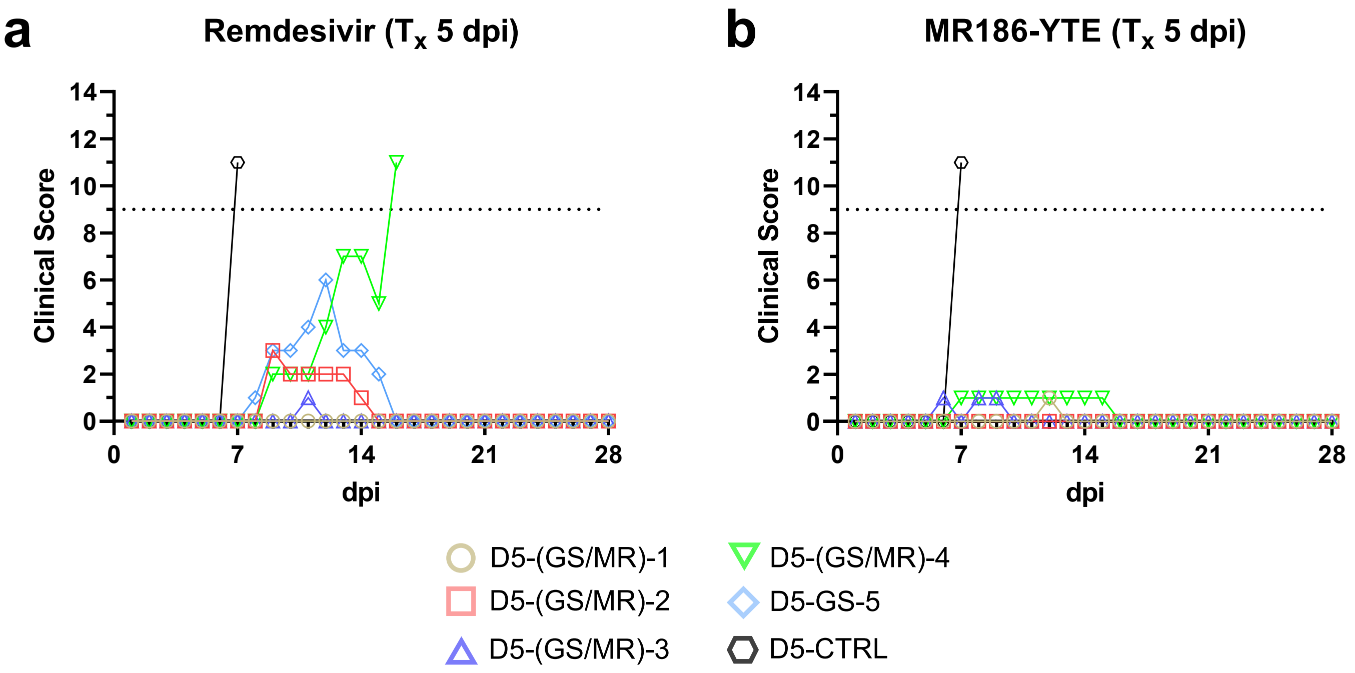

Supplementary Figure 1: Clinical scoring of MARV-challenged rhesus macaques receiving therapeutic treatment beginning 5 dpi. (a) remdesivir, **(b)** MR186-YTE. The horizontal dashed line represents the minimum clinical score by which euthanasia criteria was met. $T_{x\,=}$ “treatment”. Source data are provided as a Source Data file.

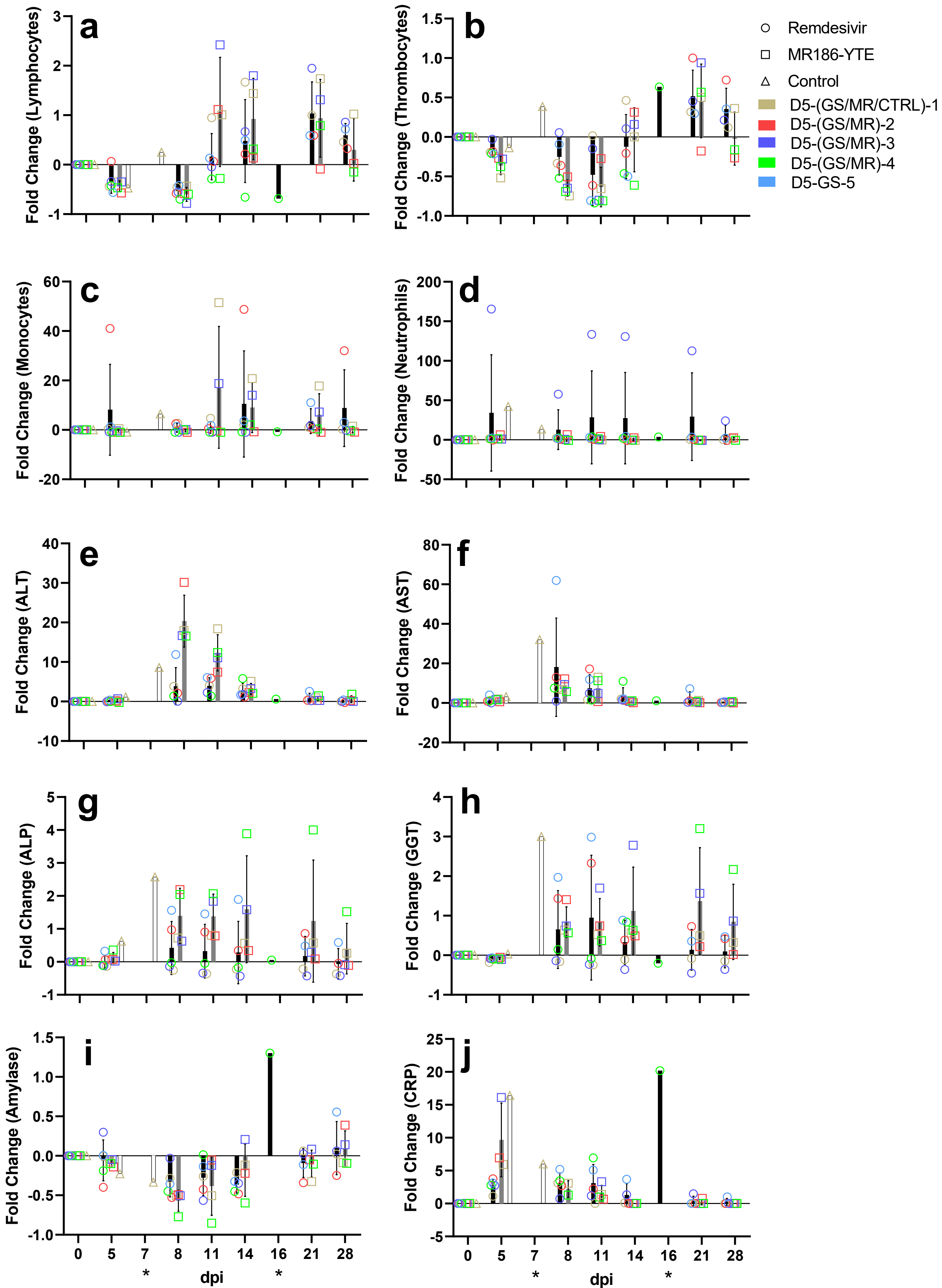

a
b
c
d
e
f
g
h
i
j
Fold Change (Lymphocytes)
Fold Change (Thrombocytes)
Fold Change (Monocytes)
Fold Change (Neutrophils)
Fold Change (ALT)
Fold Change (AST)
Fold Change (ALP)
Fold Change (GGT)
Fold Change (Amylase)
Fold Change (CRP)
Remdesivir
MR186-YTE
Control
D5-(GS/MR/CTRL)-1
D5-(GS/MR)-2
D5-(GS/MR)-3
D5-(GS/MR)-4
D5-GS-5
0 5 7 8 11 14 16 21 28
* dpi *

Supplementary Figure 2: Hematological profiles of MARV-challenged rhesus macaques receiving therapeutic treatment beginning 5 dpi. Whole blood counts and serum biochemistry analyses were performed on blood/serum obtained from each subject on the indicated days post-challenge. **(a)** lymphocytes; **(b)** thrombocytes; **(c)** monocytes; **(d)** neutrophils; **(e)** alanine aminotransferase (ALT); **(f)** aspartate aminotransferase (AST); **(g)** alkaline phosphatase (ALP); **(h)** gamma-glutamyl transferase (GGT); **(i)** amylase; **(j)** C-reactive protein (CRP). For all panels, data is presented as fold-change from individual subject baseline (0 dpi) values. Bars indicate the mean for each cohort at the indicated timepoint, error bars represent ± SD. Values for individual animals within each cohort are shown as color-coded symbols. Remdesivir treated group: n = 5 animals, MR186-YTE-treated group n = 4 animals, in-study control: n = 1 animal. Days marked with asterisks on the x-axis denote terminal timepoints for the indicated subject(s). Source data are provided as a Source Data file.

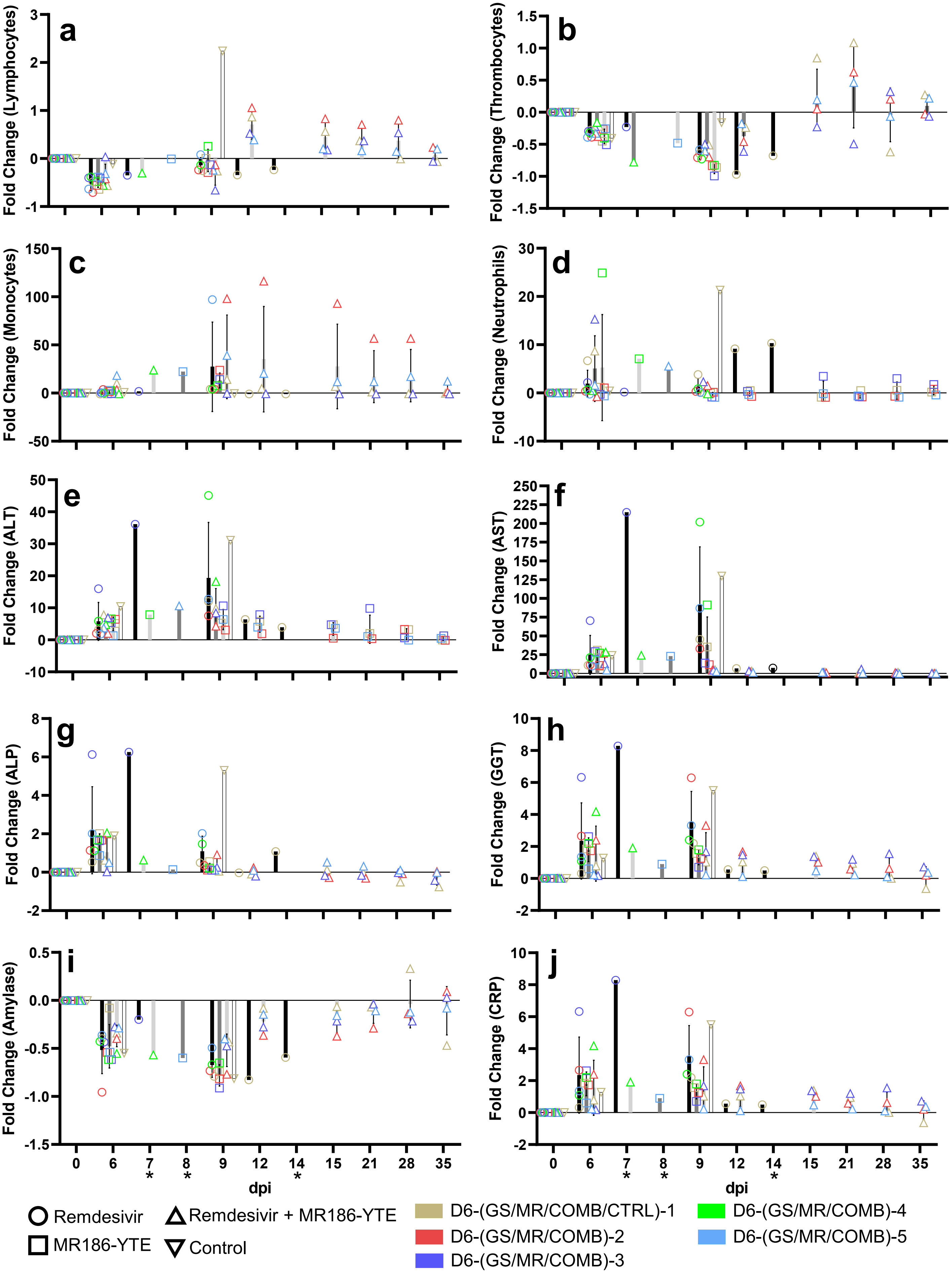

a
Fold Change (Lymphocytes)
b
Fold Change (Thrombocytes)
c
Fold Change (Monocytes)
d
Fold Change (Neutrophils)
e
Fold Change (ALT)
f
Fold Change (AST)
g
Fold Change (ALP)
h
Fold Change (GGT)
i
Fold Change (Amylase)
j
Fold Change (CRP)
0 6 7 8 9 12 14 15 21 28 35
* * *
dpi
Remdesivir
MR186-YTE
Remdesivir + MR186-YTE
Control
D6-(GS/MR/COMB/CTRL)-1
D6-(GS/MR/COMB)-2
D6-(GS/MR/COMB)-3
D6-(GS/MR/COMB)-4
D6-(GS/MR/COMB)-5

Supplementary Figure 3: Hematological profiles of MARV-challenged rhesus macaques receiving therapeutic treatment beginning 6 dpi. Whole blood counts and serum biochemistry analyses were performed on blood/serum obtained from each subject on the indicated days post-challenge. **(a)** lymphocytes; **(b)** thrombocytes; **(c)** monocytes; **(d)** neutrophils; **(e)** alanine aminotransferase (ALT); **(f)** aspartate aminotransferase (AST); **(g)** alkaline phosphatase (ALP); **(h)** gamma-glutamyl transferase (GGT); **(i)** amylase; **(j)** C-reactive protein (CRP). For all panels, data is presented as fold-change from individual subject baseline (0 dpi) values. Bars indicate the mean for each cohort at the indicated timepoint, error bars represent ± SD. Values for individual animals within each cohort are shown as color-coded symbols. Remdesivir treated group: n = 5 animals, MR186-YTE-treated group n = 5 animals, remdesivir + MR186-YTE group n = 5 animals, in-study control: n = 1 animal. Days marked with asterisks on the x-axis denote terminal timepoints for the indicated subject(s). Source data are provided as a Source Data file.

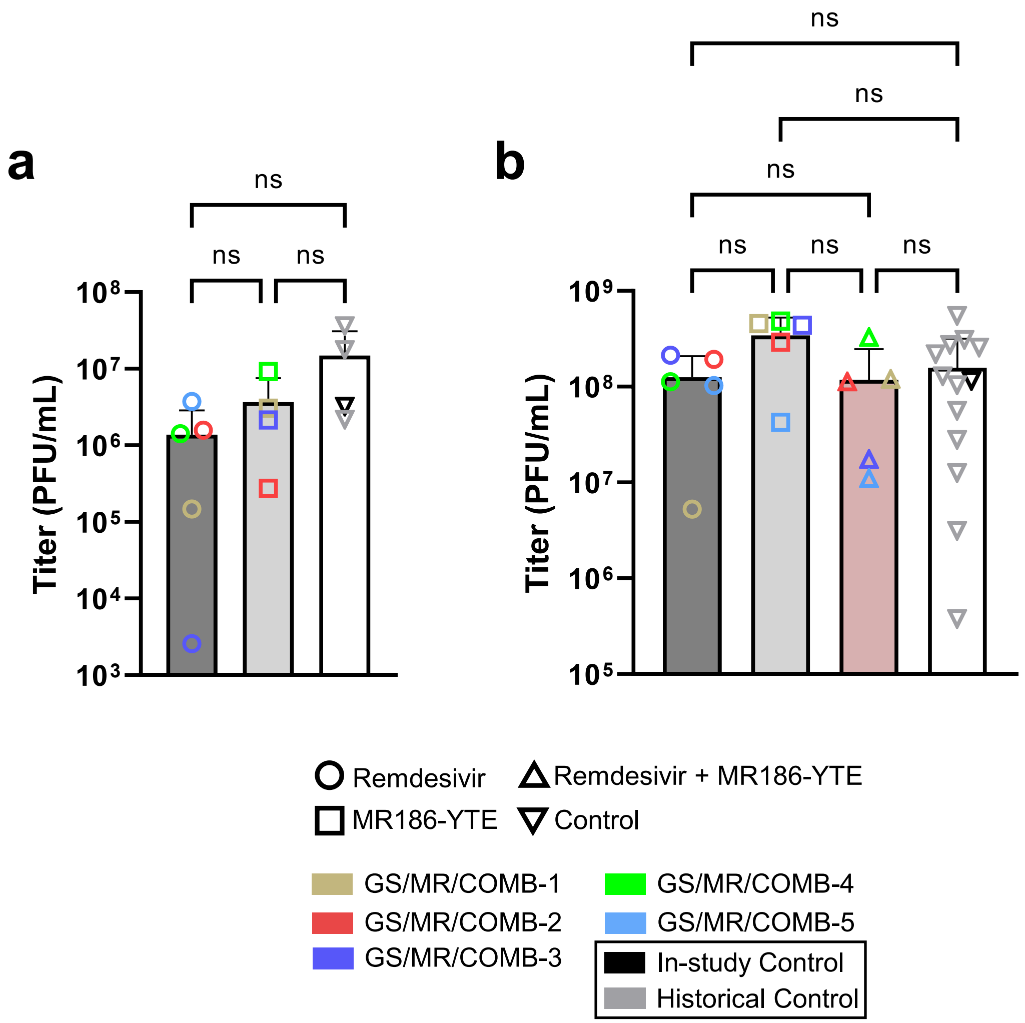

Supplementary Figure 4: Comparison of circulating viremia on day of treatment initiation between experimental and control cohorts. Plasma viremia was compared for animals from treated groups at **(a)** 5 dpi or **(b)** 6 dpi and the respective in-study control animal pooled with historical control animals sampled at the same timepoint. For **(a)**, Remdesivir treated group: n = 5 animals, MR186-YTE-treated group n = 4 animals, in-study control and historical controls: n = 4 animals. For **(b)**, Remdesivir treated group: n = 5 animals, MR186-YTE-treated group n = 5 animals, remdesivir + MR186-YTE group n = 5 animals, in-study control and historical controls: n = 13 animals. Statistical significance was assessed by 1-way ANOVA with Tukey's post-hoc test for multiple comparisons. For both timepoints, $p \geq 0.1081$ for all comparisons. ns = not significant. Source data are provided as a Source Data file.

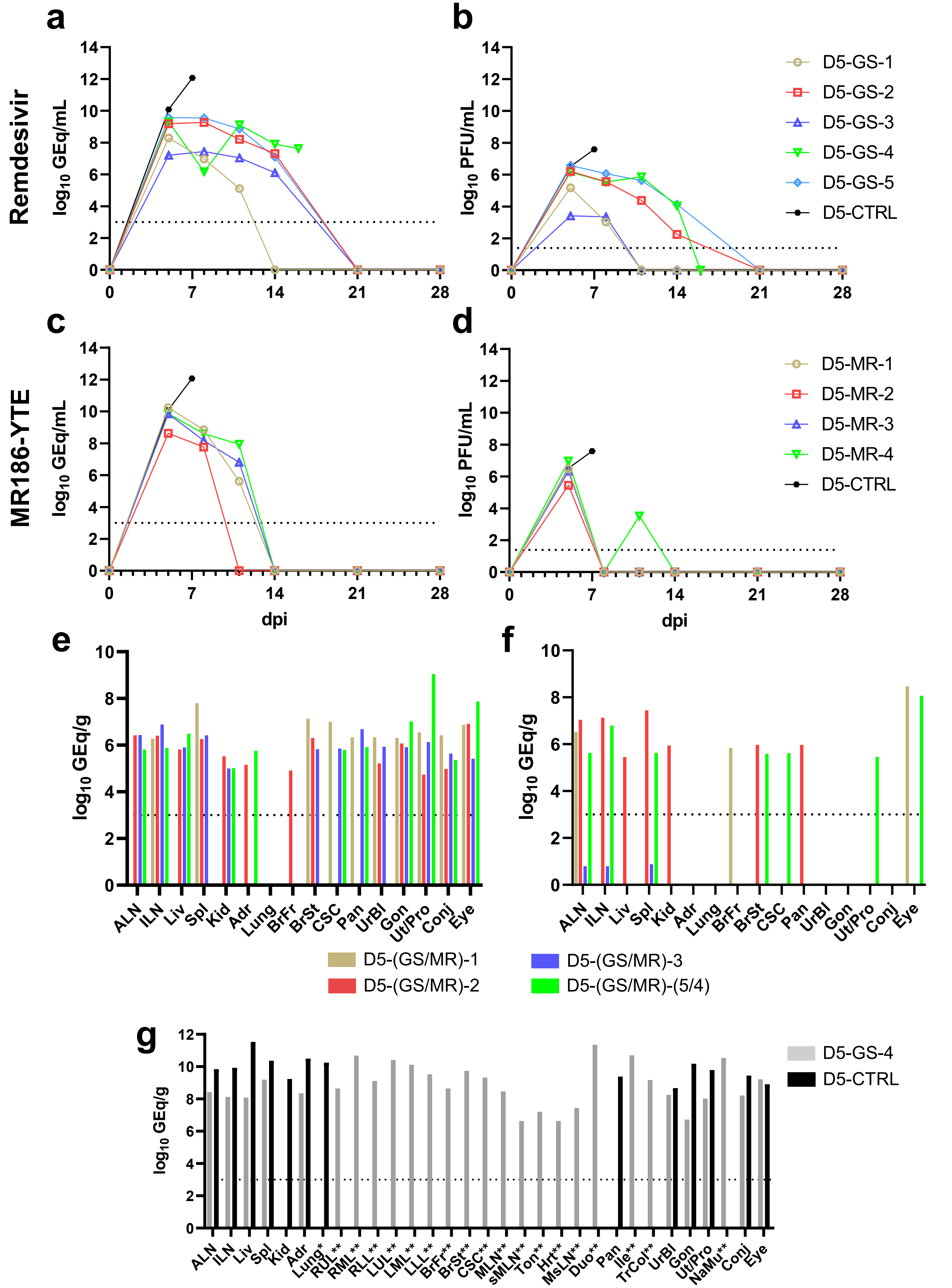
a
b
c
d
e
f
g
Remdesivir
MR186-YTE
log10 GEq/mL
log10 PFU/mL
log10 GEq/g
dpi
D5-GS-1
D5-GS-2
D5-GS-3
D5-GS-4
D5-GS-5
D5-CTRL
D5-MR-1
D5-MR-2
D5-MR-3
D5-MR-4
D5-(GS/MR)-1
D5-(GS/MR)-2
D5-(GS/MR)-3
D5-(GS/MR)-(5/4)
ALN ILN Liv Spl Kid Adr Lung BrFr BrSt CSC Pan UrBl Gon Ut/Pro Conj Eye
ALN ILN Liv Spl Kid Adr Lung* RUL** RML** RLL** LUL** LML** LLL** BrFr** BrSt** CSC** MLN** sMLN** Ton** Hrt** MsLN** Duo** Pan Ile** TrCol** UrBl Gon Ut/Pro NaMu** Conj Eye

Supplementary Figure 5: Viral load in blood and tissues of MARV-challenged rhesus macaques receiving therapeutic treatment beginning 5 dpi. Viral load was determined by RT-qPCR of whole blood (**a,c**) or selected tissues harvested at necropsy (**e-g**), or plaque titration of plasma (**b,d**). For all panels, individual data points represent the mean of two technical replicates. Bars indicate the mean for each cohort at the indicated timepoint, values for individual animals within each cohort are shown as color-coded symbols. Dashed horizontal lines indicate the limit of detection (LOD) for the assay (1000 GEq/mL for RT-qPCR; 25 PFU/mL for plaque titration). To fit on a log scale axis, zero values (below LOD) are plotted as "1" (10^0). **(e-f)** RT-qPCR analysis of tissues harvested at the study endpoint (28 dpi) from surviving animals from the remdesivir-treated **(e)** and MR186-YTE-treated **(f)** cohorts. **(g)** RT-qPCR analysis of tissues harvested from subjects D5-GS-4 and D5-CTRL, which succumbed to disease 16 and 7 dpi, respectively. For panel **(g)**, * next to the tissue name indicates that the tissue was not collected or assayed for subject D5-GS-4, and ** next to the tissue name indicate that the tissue was not collected or assayed for subject D5-CTRL. Missing data in the absence of an asterisks indicates there was a lack of detectable vRNA for that subject. Abbreviations for tissues: ALN: Axillary lymph node; ILN: inguinal lymph node; Liv: liver; Spl: spleen; Kid: kidney; Adr: adrenal gland; RUL: right upper lung; RML: right middle lung; RLL: right lower lung; LUL: left upper lung; LML: left middle lung; LLL: left lower lung; BrFr: brain frontal cortex; BrSt: brain stem; CSC: cervical spinal cord; MLN: mandibular lymph node; sMLN: submandibular lymph node; Ton: tonsil; Hrt: heart; MsLN: mesenteric lymph node; Duo: duodenum; Pan: pancreas; Ile: ileum, TrCol: transverse colon; UrBl: urinary bladder; Gon: gonad; Ut/Pro: uterus/prostate; NaMu: nasal mucosa; Conj: conjunctiva. Source data are provided as a Source Data file.

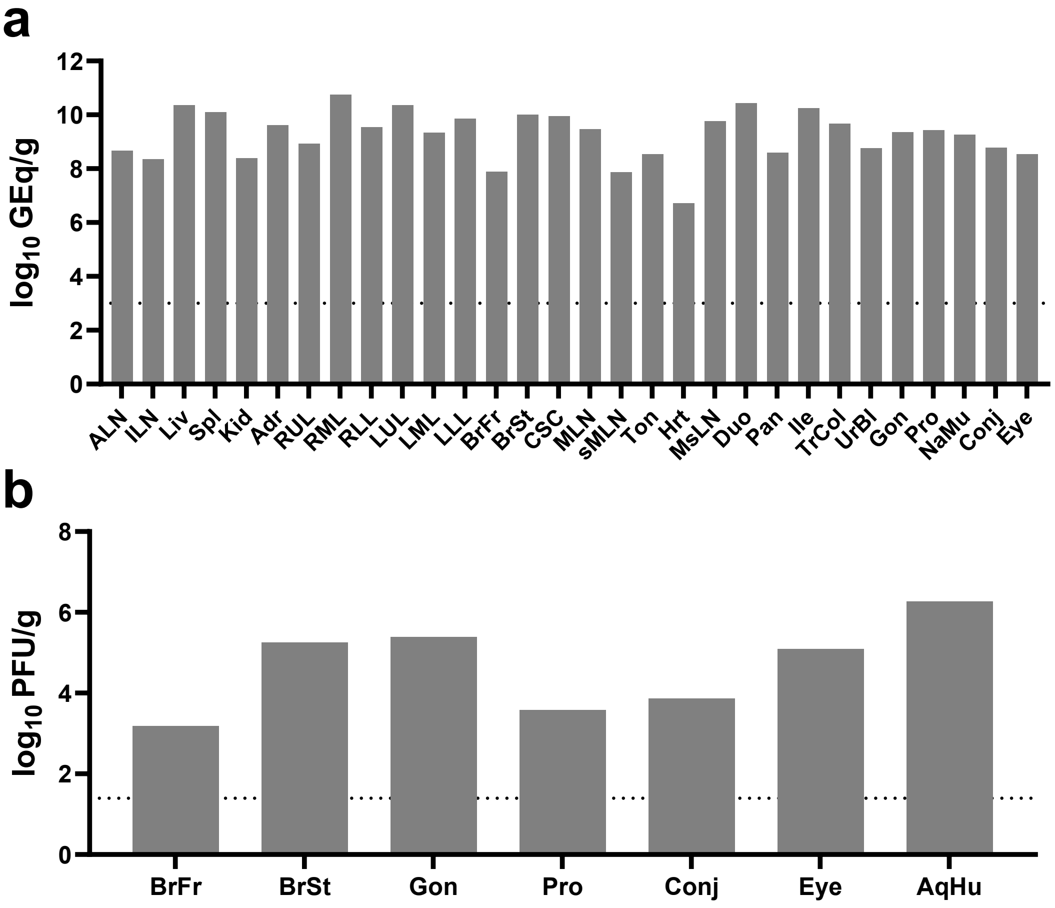

Supplementary Figure 6: Viral load in selected tissues from subject D6-GS-1. (a) RT-qPCR detection of MARV vRNA from selected tissues. **(b)** Plaque titration of infectious virus from immunologically privileged tissue sites. Dashed horizontal lines indicate the limit of detection (LOD) for the assay (1000 GEq/mL for RT-qPCR; 25 PFU/mL for plaque titration). Abbreviations for tissues: ALN: Axillary lymph node; ILN: inguinal lymph node; Liv: liver; Spl: spleen; Kid: kidney; Adr: adrenal gland; RUL: right upper lung; RML: right middle lung; RLL: right lower lung; LUL: left upper lung; LML: left middle lung; LLL: left lower lung; BrFr: brain frontal cortex; BrSt: brain stem; CSC: cervical spinal cord; MLN: mandibular lymph node; sMLN: submandibular lymph node; Ton: tonsil; Hrt: heart; MsLN: mesenteric lymph node; Duo: duodenum; Pan: pancreas; Ile: ileum, TrCol: transverse colon; UrBl: urinary bladder; Gon: gonad; Ut/Pro: uterus/prostate; NaMu: nasal mucosa; Conj: conjunctiva, AqHu: aqueous humor. Source data are provided as a Source Data file.

Supplementary Table 1. Clinical description and outcome of rhesus macaques following MARV challenge with remdesivir or MR186-YTE treatment initiated 5 dpi.

NHP	Sex	Treatment	Clinical illness	Clinical pathology
D5-GS-1	M	Remdesivir	Fever (d7); decreased appetite (d8-11, 13); flushing (d9, 11-13); inguinal lymphadenopathy (d11, 12). Subject survived to study endpoint (d28).	Lymphocytopenia (d5, 8); lymphocytosis (d14); monocytosis (d8, 11, 14); neutrophilia (d5, 8, 11, 14); eosinophilia (d5, 8, 14); basophilia (d14, 21, 28); hypoalbuminemia (d8, 11, 14); hypoamylasemia (d8, 11); CRE ↓ (d28); ALT ↑ (d8, 11, 14); AST ↑↑ (d8), ↑ (d11, 14); CRP ↑ (d5, 8)
D5-GS-2	F	Remdesivir	Fever (d7); decreased appetite (d7, 8, 12-17); anorexia (d9-11); petechial rash (d9-13); hematochezia (d9, 10); diarrhea (d11-15); hunched posture (d12-14); inguinal lymphadenopathy (d12); hypothermia (d12-14); flushing (d14). Subject survived to study endpoint (d28).	Lymphocytopenia (d8); thrombocytopenia (d8, 11); neutropenia (d14, 28); eosinopenia (d5); basopenia (d5); eosinophilia (d8); monocytosis (d5, 8, 14, 28); hypoglycemia (d21); hypoalbuminemia (d11, 14, 21, 28); hypoamylasemia (d5, 8, 11, 14, 21); ALT ↑ (d8, 14), ↑↑ (d11); AST ↑↑↑ (d8, 11), ↑ (d14, 21); GGT ↑ (d8, 11); CRP ↑ (d5, 8, 11)
D5-GS-3	M	Remdesivir	Fever (d5-10); decreased appetite (d10, 12-20); anorexia (d11); hypothermia (d16). Subject survived to study endpoint (d28).	Lymphocytopenia (d8); monocytopenia (d5, 8, 11, 14); lymphocytosis (d21); monocytosis (d21); neutrophilia (d5, 8, 11, 14, 21, 28); eosinophilia (d5, 11, 21); basophilia (d5, 14, 21, 28); hypoglycemia (d28); hypoalbuminemia (d14, 21, 28); hypoamylasemia (d11, 14); ALT ↑ (d11, 14); AST ↑ (d11, 14); CRP ↑ (d5, 11, 14, 21)
D5-GS-4	M	Remdesivir	Fever (d6-9); decreased appetite (d8, 15, 16); anorexia (d9-14); flushing (d10-14); diarrhea (d11-16); hunched posture (d12); depression (d12-16); recumbency (d13-16); weakness (14-16); hypothermia (d14-16); dyspnea (d16). Subject euthanized (d16).	Lymphocytopenia (d5, 8, 14, 16); monocytopenia (d5, 8, 11, 14, 16); eosinopenia (d16); basopenia (d11); thrombocytopenia (d8, 11, 14); neutrophilia (d5, 8, 11, 14, 16); eosinophilia (d11, 14); hyperglycemia (d16); hypoalbuminemia (d11, 14, 16); hypoamylasemia (d8, 14); hyperamylasemia (d16); BUN ↑ (d11, 14), ↑↑ (d16); CRE ↑ (d11, 14), ↑↑ (d16); ALT ↑ (d8, 11), ↑↑ (d14); AST ↑ (d5, 11, 16), ↑↑ (d8, 14); CRP ↑ (d5, 8), ↑↑ (d11), ↑↑↑ (d16)
D5-GS-5	F	Remdesivir	Fever (5-8); decreased appetite (d8, 16-21); petechial rash (d8-14); anorexia (d9-15); hunched posture (d11-15); depression (d11-13); diarrhea (d16-19). Subject survived to study endpoint (d28).	Lymphocytopenia (d5, 8); basopenia (d8); thrombocytopenia (d11, 14); monocytosis (d5, 8, 11, 14, 21, 28); neutrophilia (d5, 8, 11, 14, 21, 28); eosinophilia (d11, 14, 21, 28); basophilia (d14, 21, 28); hypoalbuminemia (d11, 14, 21, 28); hypoamylasemia (d8, 14); BUN ↑ (d11); CRE ↑ (d11), ↓ (d21, 28); ALT ↑↑ (d8, 11), ↑ (d14, 21); AST ↑ (d5, 14, 21), ↑↑↑ (d8), ↑↑ (d11); ALP ↑ (d8, 11, 14); GGT ↑ (d8, 11); CRP ↑ (5, 14, 28); ↑↑ (d8, 11)
D5-MR-1	M	MR186-YTE	Fever (d5, 8); decreased appetite (d6-11, 13); anorexia (d12). Subject survived to study endpoint (d28).	Thrombocytopenia (d5, 8, 11); lymphocytopenia (d8); neutropenia (d14, 21); eosinopenia (d14); basopenia (d8); lymphocytosis (d11, 14, 21, 28); monocytosis (d11, 14, 21, 28); hypoalbuminemia (d8, 11, 14, 21, 28); hypoamylasemia (d8, 11, 21); BUN ↓ (d8); CRE ↓ (d14); ALT ↑↑↑ (8, 11), ↑↑ (d14); AST ↑ (d5, 8, 21), ↑↑ (d11); CRP ↑↑ (d5), ↑ (d8, 11)
D5-MR-2	F	MR186-YTE	Fever (d8). Subject survived to study endpoint (d28).	Lymphocytopenia (d5, 8); monocytopenia (d5, 8, 11, 14, 21, 28); eosiniopenia (d5, 8, 14, 21); basopenia (d5, 8, 11, 21, 28); lymphocytosis (d11); neutrophilia (d5, 8, 11, 14, 28); hypoamylasemia (d8); ALT ↑↑↑↑ (d8), ↑↑ (d11), ↑ (d14); AST ↑↑↑ (d8); ALP ↑ (d8); GGT ↑ (d8); CRP ↑↑ (d5)
D5-MR-3	M	MR186-YTE	Fever (d5, 8); anorexia (d6); decreased appetite (7-11); petechial rash (d8, 9). Subject survived to study endpoint (d28).	Lymphocytopenia (d5,8); monocytopenia (d5); thrombocytopenia (d8, 11); lymphocytosis (d11, 14, 21); monocytosis (d11, 14, 21); eosinophilia (d5, 8, 21); basophilia (d5, 8, 14, 21); neutropenia (d21); hypoglycemia (d14); hypoalbuminemia (d8, 11, 14); hypoamylasemia (d8); ALT ↑↑↑ (d8, 11), ↑ (d14); AST ↑ (d5, 11), ↑↑ (d8); ALP ↑ (d11, 14); GGT ↑ (d11, 14, 21); CRP ↑↑↑ (d5), ↑ (d11)
D5-MR-4	F	MR186-YTE	Fever (d5, 8); decreased appetite (d6, 16-19); anorexia (d7-15). Subject survived to study endpoint.	Lymphocytopenia (d5, 8); monocytopenia (d5, 11, 28); neutropenia (8, 28); eosinopenia (d8); basopenia (d8, 11); thrombocytopenia (d5, 8, 11, 14); monocytosis (d14); neutrophilia (d5, 11); hypoglycemia (d11); hypoalbuminemia (d11, 14, 21); hypoproteinemia (d11); hypoamylasemia (d8, 11, 14); ALT ↑↑↑ (d8, 14), ↑ (d14, 21, 28); AST ↑ (d5, 14), ↑↑ (d8), ↑↑↑ (d11); ALP ↑ (d8, 11, 14, 21, 28); GGT ↑ (d21, 28); CRP ↑ (d8)
D5-CTRL	F	In-Study Control	Fever (d5, 6); decreased appetite (d6); anorexia (d7); depression (d7); weakness (d7); recumbency (d7);	Lymphocytopenia (d5); monocytopenia (d5); thrombocytopenia (d7); erythrocytopenia (d7); anemia (d7); monocytosis (d7); neutrophilia (d5, 7); eosinophilia (d5, 7); basopenia (d5, 7);

			petechial rash (d7), diarrhea (d7). Subject euthanized (d7).	hypoglycemia (d7); hypoalbuminemia (d7); hyamylasemia (d7); CRE ↑ (d7); ALT ↑ (d5), ↑↑ (d7); AST ↑ (d5), ↑↑↑↑ (d7); ALP ↑ (d7); GGT ↑ (d7); CRP ↑↑ (d5), ↑ (d7)

Days after MARV challenge are in parentheses. All reported findings are in comparison to baseline (d0) values, except for subject D5-MR-3, for which complete blood count (CBC) data was not available at d0 or d28 due to instrument error; pre-study data (d -50) was used to baseline comparisons. Decreased appetite is defined as some food but not all food consumed from the previous day. Anorexia is defined as no food consumed from the previous day. Fever is defined as a temperature more than 2.5 °F over baseline, or at least 1.5 °F over baseline and ≥ 103.5 °F. Hypothermia is defined as a temperature ≤3.5°F below baseline. Lymphocytopenia, monocytopenia, erythrocytopenia, thrombocytopenia, and granulocytopenia are defined by a ≥35% drop in numbers of lymphocytes, monocytes, erythrocytes, platelets, or granulocytes, respectively. Lymphocytosis, monocytosis, and granulocytosis are defined by a 100% or greater increase in numbers of lymphocytes, monocytes, or granulocytes, respectively. Hyperglycemia is defined as a 100% or greater increase in levels of glucose. Hypoglycemia is defined by a ≥25% decrease in levels of glucose. Anemia is defined as a concurrent ≥25% decrease in erythrocyte count, Hct, and Hgb. Hypoalbuminemia is defined by a ≥25% decrease in levels of albumin. Hypoproteinemia is defined by a ≥25% decrease in levels of total protein. Hypoamylasemia is defined by a ≥25% decrease in levels of serum amylase. Hypocalcemia is defined by a ≥25% decrease in levels of serum calcium. Increases in ALT, AST, ALP, CRE, CRP, Hct, and Hgb were graded on the following scale: ↑ = 1-5 fold, ↑↑ = >5-10 fold, ↑↑↑ = >10-20 fold, ↑↑↑↑ = >20-fold, ↓ = ≥50% decrease. (BUN) blood urea nitrogen, (ALT) alanine aminotransferase, (AST) aspartate aminotransferase, (ALP) alkaline phosphatase, (CRE) Creatinine, (CRP) C-reactive protein, (Hct) hematocrit, (Hgb) hemoglobin.

Supplementary Table 2. Clinical description and outcome of rhesus macaques following MARV challenge with remdesivir, MR186-YTE, or combination treatment initiated 6 dpi.

NHP	Sex	Treatment	Clinical illness	Clinical pathology
D6-GS-1	M	Remdesivir	Fever (d6-8); decreased appetite (d6, 7, 13, 14); anorexia (d8-12); petechial rash (d8-14), hematochezia (d9); periorbital edema (d10-14); ecchymosis (d11-14); hypothermia (d12, 14); hunched posture (d12-14); depression (d12-14); rhinorrhea (d14); epistaxis (d14); weakness (d14); recumbency (d14). Subject euthanized (d14).	Lymphocytopenia (d6); monocytopenia (d6, 12, 14); basopenia (d9, 12); thrombocytopenia (d9, 12, 14); monocytosis (d9); neutrophilia (d6, 9, 12, 14); eosinophilia (d6, 12, 14); ↓ Hct (d12); anemia (d9, 12, 14); hypoglycemia (d9, 12, 14); hypoalbuminemia (d9, 12, 14); hypoamylasemia (d6, 9, 12, 14); BUN ↑ (d14); CRE ↑ (d14); ALT ↑ (d6, 14), ↑↑↑ (d9), ↑↑ (d12); AST ↑↑↑ (d6), ↑↑↑↑ (d9), ↑↑ (d12, 14); ALP ↑ (d14); GGT ↑ (d9); CRP ↑↑↑↑ (d6), ↑ (d9, 12, 14)
D6-GS-2	F	Remdesivir	Fever (d6, 7); decreased appetite (d6); anorexia (d7-9); petechial rash (d7-9); hunched posture (d8, 9); jaundice (d9); depression (d8, 9); weakness (d9); recumbency (d9); hematochezia (d9). Subject euthanized (d9).	Lymphocytopenia (d6); basopenia (d6, 9); thrombocytopenia (d6, 9); monocytosis (d6, 9); anemia (d9); hypoglycemia (d9); hypoalbuminemia (d9); hypoamylasemia (d6, 9); hypoproteinemia (d9); BUN ↑ (d9); CRE ↑↑ (d9); ALT ↑ (d6), ↑↑ (d9); AST ↑↑↑ (d6), ↑↑↑↑ (d9); ALP ↑ (d6); GGT ↑ (d6), ↑↑ (d9); CRP ↑↑ (d6), ↑ (d9)
D6-GS-3	F	Remdesivir	Fever (d6); flushing (d6, 7); anorexia (d6,7); depression (d7); weakness (d7); recumbency (d7); petechial rash (d7); hypopnea (d7). Subject euthanized (d7).	Lymphocytopenia (d6); erythrocytopenia (d7); monocytosis (d7); neutrophilia (d6); eosinophilia (d7); basophilia (d7); anemia (d7); hypoglycemia (d6, 7); hypoamylasemia (d6); CRE ↑ (d7); ALT ↑↑↑ (d6), ↑↑↑↑ (d7); AST ↑↑↑↑ (d6, 7); ALP ↑↑ (d6, 7); GGT ↑↑ (d6, 7); CRP ↑↑ (d6), ↑ (d7)
D6-GS-4	M	Remdesivir	Fever (d6); reduced appetite (6, 9); anorexia (d7, 8); petechial rash (d8, 9); periorbital edema (d9); hematochezia (d9); depression (d9); recumbency (d9); unresponsiveness (d9). Subject euthanized (d9).	Lymphocytopenia (d6); basopenia (d6, 9); thrombocytopenia (d9); monocytosis (d9); hypoglycemia (d6, 9); hypoalbuminemia (d9); hypoproteinemia (d9); hypoamylasemia (d6, 9); ALT ↑↑ (d6), ↑↑↑↑ (d9); AST ↑↑↑↑ (d6, 9); ALP ↑ (d6, 9); GGT ↑ (d6, 9); CRP ↑ (d6, 9)
D6-GS-5	F	Remdesivir	Fever (d6); reduced appetite (d6-8); anorexia (d9); petechial rash (d8, 9); recumbency (d9); unresponsiveness (d9); hypopnea (d9). Subject euthanized (d9).	Lymphocytopenia (d6); eosinopenia (d6); basopenia (d6); thrombocytopenia (d6, 9); monocytosis (d9); eosinophilia (d9); hypoglycemia (d6, 9); hypoalbuminemia (d9); hypoproteinemia (d9); hypoamylasemia (d6, 9); BUN ↑ (d9); CRE ↑ (d9); ALT ↑ (d6), ↑↑↑ (d9); AST ↑↑ (d6), ↑↑↑↑ (d9); ALP ↑ (d6, 9); GGT ↑ (d6, 9); CRP ↑ (d6)
D6-MR-1	M	MR186-YTE	Reduced appetite (d6, 8, 9); emesis (d6); anorexia (d7); petechial rash (d8, 9); hunched posture (d8); jaundice (d9); depression (d9); weakness (d9); recumbency (d9). Subject euthanized (d9).	Lymphocytopenia (d6); monocytopenia (d6); thrombocytopenia (d6, 9); monocytosis (d9); neutrophilia (d6); eosinophilia (d6, 9); basophilia (d6); hypoglycemia (d6, 9); hypoalbuminemia (d9); hypoproteinemia (d9); hypoamylasemia (d9); BUN ↑ (d9); CRE ↑ (d9); ALT ↑↑ (d6, 9); AST ↑↑↑↑ (d6, 9); ALP ↑ (d6); GGT ↑ (d6, 9); CRP ↑ (d6, 9)
D6-MR-2	F	MR186-YTE	Fever (d6); reduced appetite (d6, 8, 9); anorexia (d7); petechial rash (d8, 9); jaundice (d9); depression (d9); weakness (d9); recumbency (d9). Subject euthanized (d9).	Lymphocytopenia (d6, 9); neutropenia (d6); thrombocytopenia (d6, 9); monocytosis (d6, 9); neutrophilia (d9); eosinophilia (d6, 9); anemia (d9); hypoglycemia (d9); hypoalbuminemia (d9); hypoproteinemia (d9); hypoamylasemia (d6, 9); ALT ↑ (d6, 9); AST ↑↑↑ (d6, 9); ALP ↑ (d6); GGT ↑ (d6, 9); CRP ↑↑ (d6), ↑ (d9)
D6-MR-3	M	MR186-YTE	Fever (d6); anorexia (d7); reduced appetite (d8, 9); jaundice (d9); petechial rash (d9); hematochezia (d9); depression (d9); weakness (d9); recumbency (d9). Subject euthanized (d9).	Lymphocytopenia (d6); monocytopenia (d6); basopenia (d9); thrombocytopenia (d6, 9); monocytosis (d9); neutrophilia (d6, 9); eosinophilia (d6, 9); basophilia (d6); hypoglycemia (d9); hypoalbuminemia (d9); hypoproteinemia (d9); hypoamylasemia (d6, 9); ALT ↑↑ (d6, 9); AST ↑↑↑↑ (d6), ↑↑↑ (d9); ALP ↑ (d6); GGT ↑ (d6); CRP ↑ (d9)
D6-MR-4	M	MR186-YTE	Emesis (d1, 6); fever (d6); reduced appetite (d6, 7); petechial rash (d6-9); anorexia (d8, 9); hunched posture (d8); jaundice (d9); depression (d9); recumbency (d9); unresponsiveness (d9); hypopnea (d9). Subject euthanized (d9).	Lymphocytopenia (d6); thrombocytopenia (d6, 9); monocytosis (d6, 9); eosinophilia (d6, 9); anemia (d9); hypoglycemia (d6, 9); hypoalbuminemia (d9); hypoproteinemia (d9); hypoamylasemia (d6, 9); BUN ↑ (d9); CRE ↑ (d9); ALT ↑ (d6), ↑↑↑ (d9); AST ↑↑↑↑ (d6, 9); ALP ↑ (d6); GGT ↑ (d6, 9); CRP ↑↑ (d6), ↑ (d9)
D6-MR-5	F	MR186-YTE	Fever (d6); anorexia (d6-8); emesis (d6); petechial rash (d6-8); depression (d8); weakness (d8); recumbency (d8). Subject euthanized (d8).	Lymphocytopenia (d6); thrombocytopenia (d6, 8); monocytosis (d6, 8); neutrophilia (d6, 8); eosinophilia (d6, 8); basophilia (d6, 8); hypoglycemia (d6, 8); hypoalbuminemia (d8); hypoproteinemia (d8); hypoamylasemia (d6, 8); BUN ↑ (d8), CRE ↑ (d8); ALT ↑ (d6), ↑↑↑ (d8); AST ↑↑↑ (d6), ↑↑↑↑ (d8); CRP ↑ (d8)

D6-COMB-1	M	Remdesivir + MR186-YTE	Fever (d6); decreased appetite (d7-10). Subject survived to study endpoint (d35).	Lymphocytopenia (d6); neutrocytopenia (d15); eosinopenia (d9); basopenia (d9); thrombocytopenia (d9, 28); monocytosis (d6, 9, 12, 15); neutrophilia (d6); eosinophilia (d6, 12, 21, 28, 35); basophilia (d6, 15, 21, 35); thrombocytosis (d28); hypoglycemia (d35); hypocalcemia (d35); hypoalbuminemia (d9, 12, 15, 21, 35); hypoproteinemia (d35); hypoamylasemia (d6, 9, 35); BUN ↓ (d35); ALT ↑↑ (d6, 9, 12), ↑ (d14, 21, 28); AST ↑↑ (d6), ↑ (d9, 12, 15, 28); ALP ↓ (d28, 35); GGT ↑ (d9, 12, 15), ↓ (d35); CRP ↑↑↑ (d6), ↑ (d9)
D6-COMB-2	F	Remdesivir + MR186-YTE	Fever (d6); reduced appetite (d7-11, 13); petechial rash (d7-10). Subject survived to study endpoint (d35).	Lymphocytopenia (d6); neutropenia (d12, 15, 21, 28); eosinopenia (d9, 15); basopenia (d9); thrombocytopenia (d6, 9, 12); lymphocytosis (d12); monocytosis (d6, 9, 12, 15, 21, 28, 35); hypoalbuminemia (d9, 12, 15, 21, 35); hypoamylasemia (d6, 9, 12, 15, 21); ALT ↑↑ (d6), ↑ (d9, 12, 28); AST ↑↑↑↑ (d6), ↑ (d9, 12, 28); GGT ↑ (d6, 9, 12, 15); CRP ↑↑ (d6), ↑ (d9)
D6-COMB-3	F	Remdesivir + MR186-YTE	Fever (d6, 7); reduced appetite (d6, 7, 9); anorexia (d8). Subject survived to study endpoint (d35).	Lymphocytopenia (d9); monocytopenia (d6, 9, 12, 15, 21, 28, 35); neutropenia (d9, 21); eosinopenia (d9); basopenia (d6, 9, 12, 15); thrombocytopenia (d9, 12, 21); neutrophilia (d15, 28, 35); eosinophilia (d28); hypoglycemia (d6, 9, 12, 15, 35); hypoalbuminemia (d9, 12, 15, 28); hypoamylasemia (d6, 9, 12); CRE ↓ (d28, 35); ALT ↑↑ (d6, 12, 21), ↑↑↑ (d9), ↑ (d15, 35); AST ↑↑↑ (d6), ↑ (d9, 12, 15), ↑↑ (d21); GGT ↑ (d9, 12, 15, 21, 28); CRP ↑↑ (d6), ↑ (d9)
D6-COMB-4	M	Remdesivir + MR186-YTE	Reduced appetite (d6, 7); emesis (d6); petechial rash (d7); depression (d7); weakness (d7); recumbency (d7); hematochezia (d7). Subject euthanized (d7).	Lymphocytopenia (d6); monocytopenia (d6); basopenia (d7); erythrocytopenia (d7); thrombocytopenia (d7); monocytosis (d7); neutrophilia (d6, 7); eosinophilia (d6); basophilia (d6); anemia (d7); hypoglycemia (d6, 7); hypoalbuminemia (d7); hypoproteinemia (d7); hypoamylasemia (d6, 7); BUN ↑ (d7); CRE ↑ (d7); ALT ↑↑ (d6, 7); AST ↑↑↑↑ (d6, 7); ALP ↑ (d6); GGT ↑ (d6, 7); CRP ↑↑ (d6), ↑ (d7)
D6-COMB-5	M	Remdesivir + MR186-YTE	Fever (d6-8); reduced appetite (d7-9). Subject survived to study endpoint (d35).	Neutropenia (d6, 9, 21, 28, 35); basopenia (d9); thrombocytopenia (d9); monocytosis (d6, 9, 12, 15, 21, 28, 35); hypoamylasemia (d6, 9); CRE ↓ (d21); ALT ↑ (d6, 12, 15, 21), ↑↑ (d9); AST ↑ (d6, 9, 12, 15); CRP ↑ (d6)
D6-CTRL	F	In-Study Control	Fever (d6); reduced appetite (d7, 8); anorexia (d9); petechial rash (d9); periorbital edema (d9); jaundice (d9); hunched posture (d9); depression (d9); weakness (d9); recumbency (d9). Subject euthanized (d9).	Monocytopenia (d9); thrombocytopenia (d6); lymphocytosis (d9); neutrophilia (d9); eosinophilia (d9); basophilia (d9); hypoglycemia (d6, 9); hypoalbuminemia (d9); hypoamylasemia (d6, 9); ALT ↑↑↑ (d6), ↑↑↑↑ (d9); AST ↑↑↑↑ (d6, 9); ALP ↑ (d6), ↑↑ (d9); GGT ↑ (d6), ↑↑ (d9); CRP ↑↑ (d6), ↑↑↑↑ (d9)

Days after MARV challenge are in parentheses. All reported findings are in comparison to baseline (d0) values. Decreased appetite is defined as some food but not all food consumed from the previous day. Anorexia is defined as no food consumed from the previous day. Fever is defined as a temperature more than 2.5 °F over baseline, or at least 1.5 °F over baseline and ≥ 103.5 °F. Hypothermia is defined as a temperature ≤3.5°F below baseline. Lymphocytopenia, monocytopenia, erythrocytopenia, thrombocytopenia, and granulocytopenia are defined by a ≥35% drop in numbers of lymphocytes, monocytes, erythrocytes, platelets, or granulocytes, respectively. Lymphocytosis, monocytosis, and granulocytosis are defined by a 100% or greater increase in numbers of lymphocytes, monocytes, or granulocytes, respectively. Hyperglycemia is defined as a 100% or greater increase in levels of glucose. Hypoglycemia is defined by a ≥25% decrease in levels of glucose. Anemia is defined as a concurrent ≥25% decrease in erythrocyte count, Hct, and Hgb. Hypoalbuminemia is defined by a ≥25% decrease in levels of albumin. Hypoproteinemia is defined by a ≥25% decrease in levels of total protein. Hypoamylasemia is defined by a ≥25% decrease in levels of serum amylase. Hypocalcemia is defined by a ≥25% decrease in levels of serum calcium. Increases in ALT, AST, ALP, CRE, CRP, Hct, and Hgb were graded on the following scale: ↑ = 1-5 fold, ↑↑ = >5-10 fold, ↑↑↑ = >10-20 fold, ↑↑↑↑ = >20-fold, ↓ = ≥50% decrease. (BUN) blood urea nitrogen, (ALT) alanine aminotransferase, (AST) aspartate aminotransferase, (ALP) alkaline phosphatase, (CRE) Creatinine, (CRP) C-reactive protein, (Hct) hematocrit, (Hgb) hemoglobin.

Supplementary Table 3: Histology and immunohistochemical severity scores for selected tissues from rhesus macaques challenged with MARV and treated 5 dpi

	Control	Remdesivir					MR186-YTE			
**Animal ID**	**D5-CTRL**	**D5-GS-1**	**D5-GS-2**	**D5-GS-3**	**D5-GS-4**	**D5-GS-5**	**D5-MR-1**	**D5-MR-2**	**D5-MR-3**	**D5-MR-4**
	7	28 dpi	28 dpi	28 dpi	16 dpi	28 dpi	28 dpi	28 dpi	28 dpi	28 dpi
axillary lymph node	1 1	0 0	0 0	0 0	0 0	0 0	0 0	0 0	0 0	0 0
inguinal lymph node	1 2	0 0	0 0	0 0	0 0	0 0	0 0	0 0	0 0	0 0
liver	3 3	1 0	1 0	1 0	1 1	1 0	1 0	1 0	1 0	1 0
spleen	3 3	0 0	0 0	0 0	0 1	0 0	0 0	0 0	0 0	0 0
kidney	1 1	0 0	0 0	0 0	1 1	0 0	0 0	0 0	0 0	0 0
adrenal gland	1 1	0 0	0 0	0 0	0 1	0 0	0 0	0 0	0 0	0 0
Lung	1 1	0 0	0 0	0 0	1 1	0 0	0 0	0 0	0 0	0 0
brain-cerebrum	NA	0 0	0 0	0 0	2 2	0 0	0 0	0 0	0 0	0 0
brain-brainstem/cerebellum	NA	0 0	0 0	0 0	2 2	0 0	0 0	0 0	0 0	0 0
brain-hippocamus	NA	0 0	0 0	0 0	2 2	0 0	0 0	0 0	0 0	0 0
brain-cervical spinal cord	NA	0 0	0 0	0 0	0 0	0 0	0 0	0 0	0 0	0 0
brain-pit. Gland	NA	0 0	0 0	0 0	0 0	0 0	0 0	0 0	0 0	0 0
pancreas	1 1	0 0	0 0	0 0	2 2	0 0	0 0	0 0	0 0	0 0
Urinary Bladder	1 1	0 0	0 0	0 0	1 1	0 0	0 0	0 0	0 0	0 0
gonad	0 1	0 0	0 0	0 0	1 1	0 0	0 0	0 0	0 0	0 0
Male/Female	**F**	**M**	**F**	**M**	**M**	**F**	**M**	**F**	**M**	**F**
uterus/prostate	0 1	0 0	0 0	0 0	0 1	0 0	0 0	0 0	0 0	0 0
conjunctiva/eyelids	1 1	0 0	0 0	0 0	0 1	0 0	0 0	0 0	0 0	0 0
eye	1 1	0 0	0 0	0 0	1 1	0 0	0 1	0 1	0 1	0 1

Relative severity scores of histopathological effect and detection of MARV antigen were based on the following scale for each section of tissue analyzed: 0- no lesions or immunoreactivity, 1- mild lesions or immunoreactivity (<25%), 2- moderate lesions or immunoreactivity (26-50%), 3- marked lesions or immunoreactivity (51-75%), 4- severe lesions or immunoreactivity (>75%). "NA" indicates no tissue analyzed.

First score=H&E
Second score=MARV IHC
NA: Tissue not collected/analyzed

Supplementary Table 4: Histology and immunohistochemical severity scores for selected tissues from rhesus macaques challenged with MARV and treated 6 dpi

**Group**	**Control**	**Remdesivir**					**COMBO (Remdesivir + MR186-YTE)**				
**Animal ID**	**D6-CTRL**	**D6-GS-1**	**D6-GS-2**	**D6-GS-3**	**D6-GS-4**	**D6-GS-5**	**D6-COMBO-1**	**D6-COMBO-2**	**D6-COMBO-3**	**D6-COMBO-4**	**D6-COMBO-5**
	9 dpi	14 dpi	9 dpi	7 dpi	9 dpi	9 dpi	35 dpi	35 dpi	35 dpi	7 dpi	35 dpi
Axillary lymph node	2 2	0 0	1 1	2 2	1 1	1 1	0 0	0 0	0 0	2 2	0 0
Inguinal lymph node	2 2	0 0	2 2	2 2	1 1	1 1	0 0	0 0	0 0	2 2	0 0
Liver	3 3	1 1	2 2	3 3	2 2	2 2	1 0	1 0	1 0	3 3	1 0
Spleen	3 3	1 1	3 3	3 3	2 2	2 2	0 0	0 0	0 0	3 3	0 0
Kidney	1 2	0 1	1 1	1 1	0 1	0 1	0 0	0 0	1 0	1 2	1 0
Adrenal gland	1 2	1 1	2 2	1 1	1 1	1 1	0 0	0 0	0 0	1 1	0 0
Lung	1 1	1 1	1 1	1 1	1 1	1 1	0 0	0 0	0 0	1 1	0 0
Brain-cerebrum	NA	2 2	NA	NA	NA	NA	2 1	0 0	0 0	NA	0 0
Brain-brainstem/cerebellum	NA	2 2	NA	NA	NA	NA	0 0	0 0	0 0	NA	0 0
Brain-hippocamus	NA	2 2	NA	NA	NA	NA	0 0	0 0	1 0	NA	0 0
Brain-cervical spinal cord	NA	2 2	NA	NA	NA	NA	0 0	0 0	0 0	NA	0 0
Brain-pituitary Gland	NA	0 0	NA	NA	NA	NA	0 0	0 0	0 0	NA	0 0
Pancreas	2 2	1 1	0 0	1 1	1 1	0 0	0 0	0 0	0 0	1 1	0 0
Urinary Bladder	1 2	0 0	1 1	0 0	1 1	1 1	0 0	0 0	0 0	1 2	0 0
Gonad	1 1	1 1	1 1	1 1	1 1	1 1	0 0	0 0	0 0	1 1	0 0
Male/Female	**F**	**M**	**F**	**F**	**M**	**F**	**M**	**F**	**F**	**M**	**M**
Uterus/prostate	1 1	1 1	1 1	1 1	0 0	1 1	0 0	0 0	0 0	1 1	0 0
Conjunctiva/eyelids	1 1	1 1	1 1	1 1	1 1	1 1	0 0	0 0	0 0	1 1	0 0
Eye	1 1	2 2	1 1	1 1	1 1	1 1	0 0	0 0	0 0	1 1	0 0

Relative severity scores of histopathological effect and detection of MARV antigen were based on the following scale for each section of tissue analyzed: 0- no lesions or immunoreactivity, 1- mild lesions or immunoreactivity (<25%), 2- moderate lesions or immunoreactivity (26-50%), 3- marked lesions or immunoreactivity (51-75%), 4- severe lesions or immunoreactivity (>75%). “NA” indicates no tissue analyzed.

First score=H&E
Second score=MARV IHC
NA: Tissue not collected/analyzed

Supplementary Table 4 (cont)

Group	MR186-YTE				
**Animal ID**	**D6-MR-1**	**D6-MR-2**	**D6-MR-3**	**D6-MR-4**	**D6-MR-5**
	9 dpi	9 dpi	9 dpi	9 dpi	8 dpi
Axillary lymph node	1 1	1 1	1 1	1 1	1 1
Inguinal lymph node	1 1	1 1	1 1	1 1	1 1
Liver	3 3	2 2	2 2	3 3	2 2
Spleen	3 3	2 2	2 2	3 3	2 2
Kidney	1 2	1 1	1 1	1 1	1 1
Adrenal gland	2 2	1 1	1 1	1 1	1 1
Lung	1 1	1 1	1 1	1 1	1 1
Brain-cerebrum	NA	NA	NA	NA	NA
Brain-brainstem/cerebellum	NA	NA	NA	NA	NA
Brain-hippocamus	NA	NA	NA	NA	NA
Brain-cervical spinal cord	NA	NA	NA	NA	NA
Brain-pituitary Gland	NA	NA	NA	NA	NA
Pancreas	1 1	0 0	1 1	1 1	1 1
Urinary Bladder	1 1	0 0	1 1	1 1	1 1
Gonad	1 1	1 1	1 1	1 1	1 1
Male/Female	**M**	**F**	**M**	**M**	**F**
Uterus/prostate	1 1	1 1	1 1	1 1	1 1
Conjunctiva/eyelids	1 1	1 1	1 1	1 1	1 1
Eye	1 1	1 1	1 1	1 1	1 1

Relative severity scores of histopathological effect and detection of MARV antigen were based on the following scale for each section of tissue analyzed: 0- no lesions or immunoreactivity, 1- mild lesions or immunoreactivity (<25%), 2- moderate lesions or immunoreactivity (26-50%), 3- marked lesions or immunoreactivity (51-75%), 4- severe lesions or immunoreactivity (>75%). "NA" indicates no tissue analyzed.

First score=H&E
Second score=MARV IHC
NA: Tissue not collected/analyzed

Supplementary Table 5: MARV Angola *NP* RT-qPCR detection primers

Primer Name	Primer Sequence
MARV Angola *NP* Forward	5’-CAG GAT CCC TTT GGC AGT TT-3’
MARV Angola *NP* Reverse	5’-TAG GCT TCT CTT GCC CTT GT-3’
MARV Angola *NP* Probe	6-carboxyfluorescein (6FAM)-5′-CCC ATA AGG TCA CCC TCT T-3′-carboxytetramethylrhodamine (TAMRA)
